# Comprehensive ECG reference intervals in C57BL/6N substrains provide a generalizable guide for cardiac electrophysiology studies in mice

**DOI:** 10.1007/s00335-023-09995-y

**Published:** 2023-06-09

**Authors:** Manuela A. Oestereicher, Janine M. Wotton, Shinya Ayabe, Ghina Bou About, Tsz Kwan Cheng, Jae-Hoon Choi, Dave Clary, Emily M. Dew, Lahcen Elfertak, Alain Guimond, Hamed Haseli Mashhadi, Jason D. Heaney, Lois Kelsey, Piia Keskivali-Bond, Federico Lopez Gomez, Susan Marschall, Michael McFarland, Hamid Meziane, Violeta Munoz Fuentes, Ki-Hoan Nam, Zuzana Nichtová, Dale Pimm, Lynette Bower, Jan Prochazka, Jan Rozman, Luis Santos, Michelle Stewart, Nobuhiko Tanaka, Christopher S. Ward, Amelia M. E. Willett, Robert Wilson, Robert E. Braun, Mary E. Dickinson, Ann M. Flenniken, Yann Herault, K. C. Kent Lloyd, Ann-Marie Mallon, Colin McKerlie, Stephen A. Murray, Lauryl M. J. Nutter, Radislav Sedlacek, Je Kyung Seong, Tania Sorg, Masaru Tamura, Sara Wells, Elida Schneltzer, Helmut Fuchs, Valerie Gailus-Durner, Martin Hrabe de Angelis, Jacqueline K. White, Nadine Spielmann

**Affiliations:** 1 Institute of Experimental Genetics and German Mouse Clinic, Helmholtz Center Munich, German Research Center for Environmental Health, Ingolstädter Landstraße 1, 85764 Neuherberg, Germany; 2 The Jackson Laboratory, 600 Main Street, Bar Harbor, ME 04609 USA; 3 Experimental Animal Division, RIKEN BioResource Research Center, 3-1-1 Koyadai, Tsukuba, Ibaraki 305-0074 Japan; 4 Université de Strasbourg, CNRS, INSERM, Institut de La Clinique de La Souris, PHENOMIN, 1 Rue Laurent Fries, 67404 Illkirch, France; 5 The Mary Lyon Centre, MRC Harwell, Harwell Campus, Oxfordshire, OX11 0RD UK; 6 European Molecular Biology Laboratory, European Bioinformatics Institute, Wellcome Trust Genome Campus, Hinxton, Cambridgeshire, CB10 1SD UK; 7 Molecular and Human Genetics, Baylor College of Medicine, One Baylor Plaza, Houston, TX 77030 USA; 8 The Centre for Phenogenomics, Lunenfeld-Tanenbaum Research Institute, Mount Sinai Hospital, Toronto, ON M5T 3H7 Canada; 9 The Centre for Phenogenomics, The Hospital for Sick Children, Toronto, ON M5T 3H7 Canada; 10 Integrated Bioresource Information Division, RIKEN BioResource Research Center, 3-1-1 Koyadai, Tsukuba, Ibaraki 305-0074 Japan; 11 Integrative Physiology, Baylor College of Medicine, One Baylor Plaza, Houston, TX 77030 USA; 12 Department of Laboratory Medicine & Pathobiology, Faculty of Medicine, University of Toronto, Toronto, ON Canada; 13 Mouse Biology Program, University of California, 2795 Second Street Suite 400, Davis, CA 95618 USA; 14 Czech Centre for Phenogenomics, Institute of Molecular Genetics of the Czech Academy of Sciences, Prague, Czech Republic; 15 Laboratory of Developmental Biology and Genomics, College of Veterinary Medicine, and Interdisciplinary Program for Bioinformatics, Korea Mouse Phenotyping CenterBK21 Plus Program for Advanced Veterinary Science, Research Institute for Veterinary ScienceSeoul National University, 599 Gwanak-Ro, Gwanak-Gu, Seoul, 08826 Republic of Korea; 16 Technology and Development Team for Mouse Phenotype Analysis, RIKEN BioResource Research Center, 3-1-1 Koyadai, Tsukuba, Ibaraki 305-0074 Japan; 17 Chair of Experimental Genetics, School of Life Science Weihenstephan, Technische 83 Universität München, Alte Akademie 8, 85354 Freising, Germany; 18 German Center for Diabetes Research (DZD), Ingolstädter Landstrasse 1, 85764 Neuherberg, Germany; 19 Department of Life Science, College of Natural Sciences, Hanyang Institute of Bioscience and Biotechnology, Research Institute for Natural Sciences, Hanyang University, Seoul, 04763 Republic of Korea; 20 Korea Mouse Phenotyping Center, Korea Research Institute of Bioscience and Biotechnology, Daejeon, Republic of Korea

## Abstract

Reference ranges provide a powerful tool for diagnostic decision-making in clinical medicine and are enormously valuable for understanding normality in pre-clinical scientific research that uses *in vivo* models. As yet, there are no published reference ranges for electrocardiography (ECG) in the laboratory mouse. The first mouse-specific reference ranges for the assessment of electrical conduction are reported herein generated from an ECG dataset of unprecedented scale. International Mouse Phenotyping Consortium data from over 26,000 conscious or anesthetized C57BL/6N wildtype control mice were stratified by sex and age to develop robust ECG reference ranges. Interesting findings include that heart rate and key elements from the ECG waveform (RR-, PR-, ST-, QT-interval, QT corrected, and QRS complex) demonstrate minimal sexual dimorphism. As expected, anesthesia induces a decrease in heart rate and was shown for both inhalation (isoflurane) and injectable (tribromoethanol) anesthesia. In the absence of pharmacological, environmental, or genetic challenges, we did not observe major age-related ECG changes in C57BL/6N-inbred mice as the differences in the reference ranges of 12-week-old compared to 62-week-old mice were negligible. The generalizability of the C57BL/6N substrain reference ranges was demonstrated by comparison with ECG data from a wide range of non-IMPC studies. The close overlap in data from a wide range of mouse strains suggests that the C57BL/6N-based reference ranges can be used as a robust and comprehensive indicator of normality. We report a unique ECG reference resource of fundamental importance for any experimental study of cardiac function in mice.

**Supplementary Information:**

The online version contains supplementary material available at 10.1007/s00335-023-09995-y.

## Introduction

Reference ranges are a powerful tool for diagnostic decision-making in clinical medicine and their use has become increasingly common **(**Rijnbeek et al. 2001; Williams et al. 2020**)**. Reference ranges are derived intervals containing a defined subset of values from a large and comparable population dataset. These values, designed to delineate the expected range of a given parameter, are used clinically to identify outlier values. Individuals presenting with values outside of a clinically defined reference range are considered abnormal and flagged for follow up clinical investigation.

Looking beyond clinical applications, reference ranges are of enormous value in pre-clinical, basic scientific research using in vivo modelling (Otto et al. 2016). They are used to define “normality” for a given genetic background, sex, and age of animals, such as inbred mouse strains. To our knowledge, there are no published reference ranges for electrocardiography (ECG) in the laboratory mouse. Such reference ranges would provide the research community with the information necessary to evaluate the consequences of pharmacological, environmental, or genetic perturbations, the latter opening up the opportunity to uncover genotype*phenotype associations.

We used ECG data collected under the auspices of the International Mouse Phenotyping Consortium (IMPC) (Dickinson et al. 2016) (https://www.mousephenotype.org), to generate the first mouse-specific cardiac physiology reference ranges. Here, data were collected from over 26,000 conscious or anesthetized C57BL/6N wildtype control mice stratified by sex and age. The unprecedented scale of this data resource yields a robust reference range for a broad and commonly studied set of ECG parameters that are clinically important to assess myocardial electrical processes and cardiac function.

## Materials/methods

### The International Mouse Phenotyping Consortium

The International Mouse Phenotyping Consortium (IMPC) represents a multi-institutional and collaborative research initiative encompassing twenty-four major research organizations and funding agencies, distributed globally (Dickinson et al. 2016). The IMPC seeks to generate and phenotype a knockout mouse line for every protein-coding gene in the mouse genome (www.mousephenotype.org) (Muñoz-Fuentes et al. 2018). Phenotyping is carried out under the uniform operating procedures detailed in IMPReSS (International Mouse Phenotyping Resource of Standardized Screens; www.mousephenotype.org/impress/index), which were developed and validated during the pilot programs EUMORPHIA and EUMODIC (Green et al. 2005).

### IMPC centers contributing electrocardiography data

IMPC data release (DR) 15.0 was used herein (https://www.mousephenotype.org/data/previous-releases/15.0). The following subset of ten IMPC data-contributing centers provided electrocardiography (ECG) data in DR 15.0 (ethical approval details are included in parenthesis after each contributing center):

1. Baylor College of Medicine (BCM) (Institutional Animal Care and Use Committee approved license AN-5896).
2. German Mouse Clinic Helmholtz Zentrum München (GMC) (#144-10, 15-168)
3. Medical Research Council (MRC) – Harwell (HAR) (Animal Welfare and Ethical Review Body approved licenses 70/8015 and 30/3384).
4. Institute Clinique de la Souris, Mouse Clinical Institute (ICS) (#4789-2016040511578546v2).
5. The Jackson Laboratory (JAX) (Institutional Animal Care and Use Committee approved licenses 14,004, 11,005, and 99,066. JAX AAALAC accreditation number 000,096, NIH Office of Laboratory Animal Welfare assurance number D16-00,170).
6. RIKEN BioResource Research Center (RBRC) (Animal Care Committee approved animal use protocols 0153, 0275, 0277, and 0279).
7. University of California – Davis (UCD) (Institutional Animal Care and Use Committee approved animal care and use protocol number 19,075. UCD AAALAC accreditation number 000029, and the NIH Office of Laboratory Animal Welfare assurance number D16-00,272 # (A3433-01).
8. Seoul National University, Korea Mouse Phenotyping Center (KMPC) (KRIBB-AEC-19189).
9. Czech Centre for Phenogenomics (CCP) (AV CR 62/2016, Academy of Sci., Czech Rep.).
10. The Centre for Phenogenomics, Toronto (TCP) (22-0275 and 22-0279).

ECG data were collected from mice at one of two possible timepoints. For the Early Adult (EA) Pipeline, data were collected at a mean of 12 weeks with the minimum of 8 and maximum of 16 weeks of age. For the Late Adult (LA) Pipeline, data were collected at a mean of 62 weeks with the minimum of 52 and maximum of 78 weeks of age. Animal welfare was assessed routinely for all mice involved.

### Animals

This study includes data collected from inbred wildtype control animals tested as part of the IMPC goals. These mice, both males and females, were on a C57BL/6N genetic background of substrains: C57BL/6NCrl (CCP, HMGU, ICS, TCP and UCD); C57BL/6NJ (JAX and BCM); C57BL/6NJcl (RBRC) and C57BL/6NTac (KMPC, HMGU, ICS and HAR). Non-IMPC mice were from four different studies: (1) The founder strains animals from a study titled “The Collaborative Cross: A Recombinant Inbred Mouse Population for the Systems Genetic Era” (Threadgill et al. 2011) with A/J, C57BL/6J, 129S1/SvlmJ, NOD/ShiLtJ, NZO/HlLtJ, CAST/EiJ, PWK/Ph, and WSB/EiJ inbred strains (https://phenome.jax.org/projects/GMC13); (2) The Jaxwest1 project, a multi-system analysis of physiology on seven inbred strains of mice: 129S1/SvImJ, A/J, BALB/cJ, C57BL/6J, DBA/2J, NOD/ShiLtJ and SJL/J (https://phenome.jax.org/projects/Jaxwest1); (3) Wildtype control animals from three non-IMPC studies performed at the German Mouse Clinic (https://www.mouseclinic.de/) with a standard sample size (20–30 control animals per study). The mouse backgrounds were: (i) An independent repeat of strain C57BL/6NJ (Jackson Laboratory strain #:005304) that is used by some of the IMPC contributing centers; (ii) C57BL/6J (JAX strain #:000664), the most commonly used inbred mouse strain and the first to have its genome sequenced; and (iii) FVB (JAX strain #:001800), a widely used multipurpose inbred line. For more information on these inbred strains, visit: https://www.jax.org/strain; and (4) The Xing1, Aging study: Electrocardiogram for 29 inbred strains of mice (https://phenome.jax.org/projects/Xing1) (Xing et al. 2009). Xing1 recorded ECG characteristics in the following 26 inbred mouse strains: 129S1/SvImJ, A/J, BALB/cByJ, BTBR *T*^+^ *Itpr3*^*tf*^/J, BUB/BnJ, C3H/HeJ, C57BL/10J, C57BL/6J, C57BLKS/J, C57BR/cdJ, C57L/J, CBA/J, DBA/2J, FVB/NJ, KK/HIJ, LP/J, MRL/MpJ, NOD.B10Sn-*H2*^*b*^/J, NON/ShiLtJ, NZO/HlLtJ, NZW/LacJ, P/J, PL/J, RIIIS/J, SM/J, and SWR/J. AKR/J, PWD/PhJ and SJL/J were excluded herein due to incomplete ECG data.

### Data collection

The IMPC standard operating procedure provides an overview of the conscious and anesthetized ECG procedures used by contributing centers (https://www.mousephenotype.org/impress/ProcedureInfo?action=list&procID=1415&pipeID=7). In brief, conscious ECG was collected using ECGenie equipment (Mouse Specifics, Inc.) as detailed previously by Spielmann (Spielmann et al. 2022). Based on availability of equipment and local expertise, some contributing centers opted to perform anesthetized ECG using Power Lab recording equipment and LabChart8 software (ADInstruments), configured in the following way. All centers used the “Mouse” preset detection and analysis settings and the “Rodent T-wave” analysis mode. The default values in the LabChart detection and analysis settings were as follows: the typical QRS width was 10 ms; R-waves were at least 60 ms apart; the Pre-P baseline was 10 ms; the maximum PR was 50 ms; the maximum RT was 40 ms; and the ST height was measured at 10 ms from alignment. Detailed information about ECG acquisition, including these default settings and parameter analysis are available (https://www.mousephenotype.org/impress/ProcedureInfo?action=list&procID=1426).

Mice were anesthetized either with inhaled isoflurane (anesthesia was induced using 2.5–4% isoflurane in oxygen then maintained using 2–2.5% isoflurane in oxygen) or injected tribromoethanol (Sigma, stock concentration 20 mg/ml, dose calculated as 0.5 g/kg body weight). Anesthetized mice were positioned supine on a warming pad apparatus that maintained the animal’s core temperature at 37 °C. Needle electrodes were placed subcutaneously as follows: the negative electrode in the right forelimb; the ground electrode in the right hindlimb; and the positive electrode in the left hindlimb. ECG data were collected for up to 120 s and the resulting data analyzed using LabChart software (ADInstruments). Regardless of the methodology, ECG was recorded in a dimly lit, quiet procedure room. In order to eliminate circadian influences ECG was recorded during the morning when the resting phase of a mouse begins.

### Data annotation and quality

Standard protocols for ECG signal analysis were used to analyze the data. For each cardiac cycle, the P, Q, R, S and T peaks were defined and used to derive a total of fifteen parameters including intervals, amplitudes, and dispersions (Supplemental Table 1).

In both, conscious (ECGenie equipment) and anesthetized (LabChart using the preset windows stipulated above) EGC, P, Q, R, S and T peaks were automatically detected by averaging over multiple cardiac cycles. If any peaks were not selected correctly by either software, the position of this marker was corrected manually. Heart rate variability (HRV) was calculated as the mean of the differences between successive heart rates for the entire set of ECG signals. The QT-intervals were frequency corrected (QTc) by applying the following equation derived by Mitchell (Mitchell et al. 1998): QTc = [QT/√(RR/100)]*1000 s. Noise and movement artefacts were automatically eliminated by the software.

**Fig. 1:**
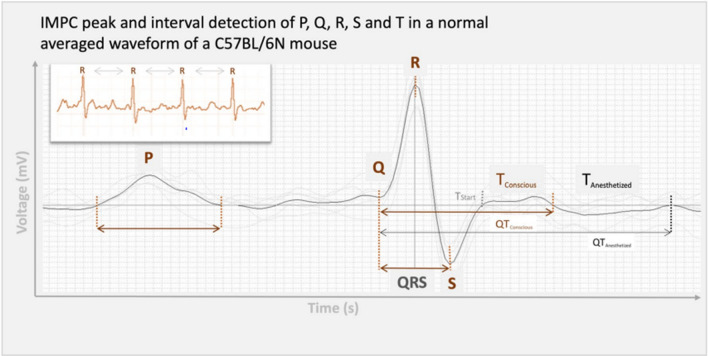
Representative averaged waveform of a C57BL/6N mouse as a function of voltage over time, reflecting the most commonly applied ECG annotations with P-, Q-, R-, S-, and T-peak detection. Some differences in the placement of S- and T-annotations between centers participating in the IMPC were observed. Differences in T-peak placement are represented as T_Conscious_ and T_Anesthetized_

Some subtle differences in wave marker placement were observed across IMPC contributing centers. P, Q and R marker locations were annotated consistently by all contributing centers. In Fig. 1, S is annotated at the peak negative inflection point of the QRS complex, which accurately reflects the majority of data reported herein, including all data collected from conscious mice. However, for datasets collected on anesthetized mice, contributing centers variably annotated S at the peak negative inflection point, the isoelectric point, or halfway between peak negative inflection and isoelectric point. In Fig. 1, T_Conscious_ is annotated as the peak positive inflection following S, which accurately reflects the majority of data reported herein, including all data collected from conscious mice. However, that peak can also be annotated as J (Calvet and Seebeck 2023). This reflects the considerable variation and controversy in mouse ECG literature around the positioning of T (Berul et al. 1996; Boukens et al. 2012; Doevendans et al. 1998; Goldbarg et al. 1968). One advantage of anesthetized ECG is that signal noise decreases as the animals are immobilized and with this decrease in electrical noise, the sensitivity of the waveform detection improves. All the centers that contributed ECG data collected from isoflurane and tribromoethanol anesthetized mice annotated the T-peak (T_Anesthetized_) as the gently sloping negative inflection after “J”. This is consistent with Calvert (Calvet and Seebeck 2023). This is a substantive difference in T-wave annotation between conscious and anesthetized animals.

Data were curated and subject to quality control at the IMPC prior to Data Release 15 (August 11th, 2021) and we excluded one additional mouse from the analysis due to a biologically implausible QRS value.

### Statistical methods

Bespoke methods were developed to assess ECG reference ranges and are independent of the methodologies implemented on the IMPC portal.

Data analysis was conducted using R [version 4.0.4, R Core Team 2022 (Team 2022)] with figures and tables produced in ggplot2, embedded in RMarkdown HTML files. Variability of all the data were assessed with two metrics (a) coefficient of variation (COV) and (b) “Quartile-based CV” (QCV), defined as interquartile range (IQR) (75–25%) relative to the median (100*IQR/median).

Visual methods, as well as formal statistical tests were applied to test whether the scores of the individual parameters were normally distributed. Data were separated by age, sex and anesthesia regime and histograms for each parameter were plotted. Shapiro-Wilks tests were conducted to assess normality. Reference ranges were calculated based on median, 25th percentile and 75th percentile. In addition, the mean, standard deviation, and parameter sample size were provided to reflect the distribution of data. To reflect the distribution of each parameter, the 95% confidence intervals can be calculated by mean ± 1.96*standard deviation for each parameter.

### Investigation of anesthesia, sex and age effects

To investigate the effect of anesthesia on the different parameters, we calculated a one-way Analysis of Variance (ANOVA) with planned comparisons of “Conscious versus Isoflurane” and “Conscious versus Tribromoethanol”, separated by sex whereas “Isoflurane versus Tribromoethanol” was not tested. These planned comparisons were used to compare conscious vs unconscious. When looking for differences between groups we tested the null hypothesis. *p*-values and *F*-values with degrees of freedom were calculated.

The effects of sex (female vs male) and age (EA vs LA) were compared using the same statistical analyses. In each case a simple two-tailed t-test was performed and the Cohen’s *d* effect size calculated from the “effsize package” (R library). Due to the central limit theorem (CLT) (Zhang et al. 2022), the large sample sizes allowed parametric statistical testing of these effects.

These large group sizes provide overwhelming statistical power and may overestimate the importance of the effects. Bootstrapping tests were done to verify the biological significance of any differences in a range of more realistic experimental group sizes.

## Results

ECG data collected by IMPC contributing centers (data release, DR, 15.0) were available from 26,706 wildtype control mice, stratified as presented in Table 1 and summarized below. All the mice were from a C57BL/6N-inbred substrain. ECG was performed on conscious mice, or mice anesthetized with either isoflurane or tribromoethanol. The majority of mice (90.6% or 24,194) were tested at a mean age of 12 weeks (designated as Early Adult or EA), while the remaining 9.4% (2512) of mice were tested at a mean age of 62 weeks (designated as Late Adult or LA). Sex was evenly distributed at both EA and LA timepoints. Raw data can be downloaded using the following link: https://www.mousephenotype.org/data/previous-releases/15.0. The total number of reported parameters varied slightly between mice and can be accessed in Supplemental Table 2

**Table 1:** ECG data were available from a total of 26,706 mice, stratified by sex, age at testing (EA = mean of 12 weeks of age; LA = mean of 62 weeks of age), and conscious state (conscious, anesthetized using isoflurane, or anesthetized using tribromoethanol)

	Conscious	Isoflurane	Tribromoethanol	Sum
EA Females	9240	2672	226	12,138
EA Males	9238	2598	220	12,056
LA Females	620	693	0	1313
LA Males	589	610	0	1199
Sum	19,687	6573	446	26,706

### Variability assessment

A panel of 15 output parameters were collected from ECG, namely heart rate (HR), RR-, PR-, PQ-, ST-, and QT-interval, and QT corrected (QTc) using the Mitchell formula^4^, QRS complex, coefficient of variation of R-R intervals (CV), heart rate variability (HRV), pNN5, rMSSD (Root Mean Sum of Squared Distance), mean R-amplitude, mean SR-amplitude and QT corrected (QTc) dispersion (parameter definition in Supplemental Table 1).

In multi-center, large-scale, high-throughput programs such as the IMPC, variability in the measured values was to be expected. However, the extent of this variability dictates the sensitivity and robustness of each parameter.

Variability testing was performed on all DR 15.0 ECG data from the IMPC, independently of anesthetic agent in this analysis. For each sex, individual ECG parameters were tested for variability in EA and LA populations. The following standard metrics for assessing distribution variability were calculated:

(1) Coefficient of variation (COV) (100*standard deviation/mean) assumes a parametric distribution and normalizes the variability to the most typical score (mean) but is sensitive to outliers. (2) To support the parametric COV test, we applied a “Quartile-based CV” (QCV), defined as interquartile range (IQR) (75–25%) relative to the median (100*IQR/median). QCV is a similar metric to COV but uses non-parametric measures of variability, therefore makes no assumptions of normality but is still readily influenced by outliers (Arachchige et al. 2022; Leys et al. 2013).

**Fig. 2:**
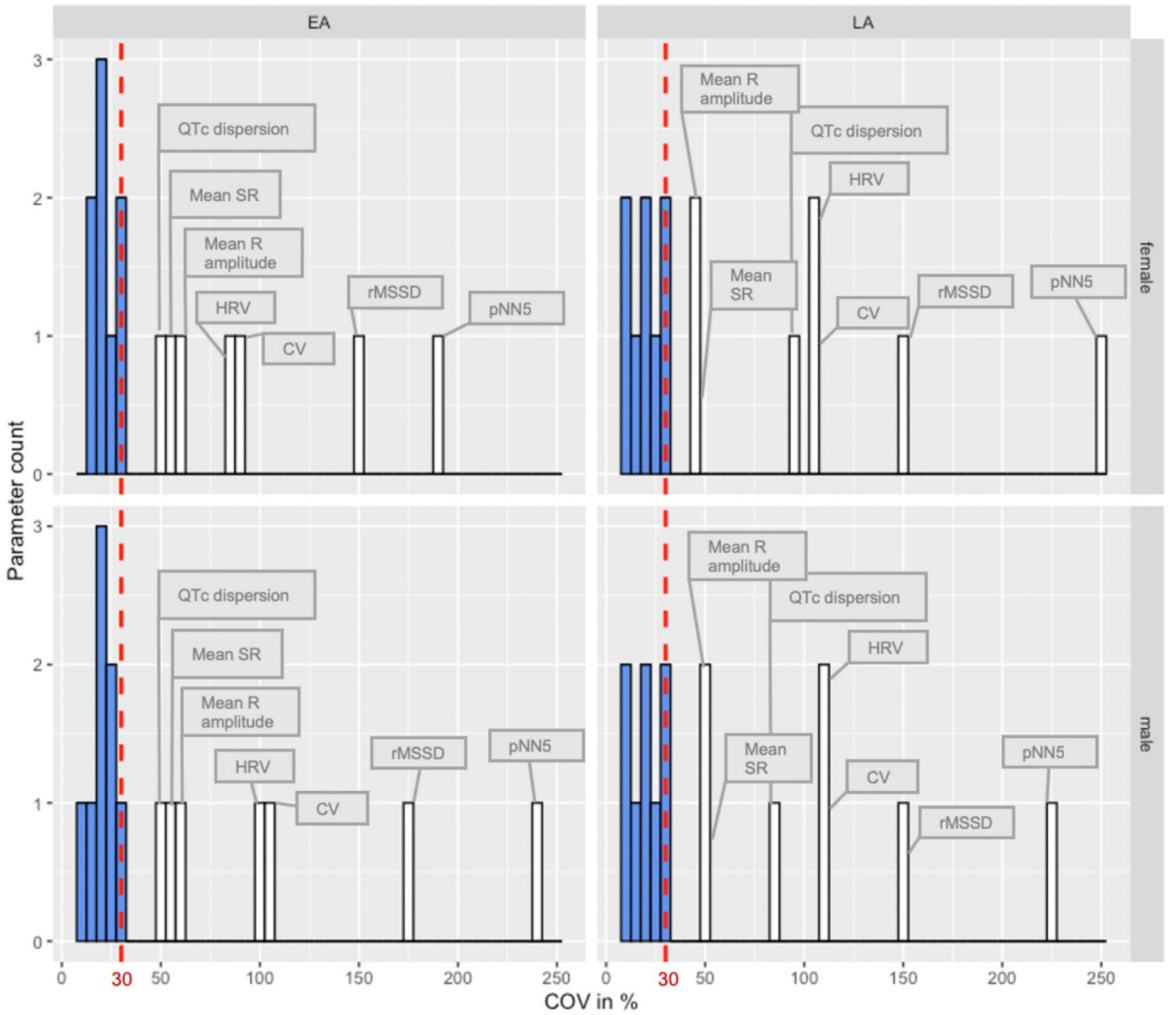
Coefficient of variation (COV) analysis of data split by sex (female and male) and age (EA and LA) identified parameters with excess variability (COV > 30%) that were excluded from further analysis (white bars). Parameters in blue were below the COV threshold of 30% and were retained for further analysis. These were in ascending COV percentage QTc Mitchell, PQ-, QT-, QRS complex, ST-, HR-, RR- and PR-interval

Based on this analysis, exclusion criteria were defined as any parameter with acceptable variability based on Eurachem guidelines (https://www.eurachem.org/index.php/publications/guides) of ≥ 30 for COV (Fig. 2) and a QCV ≥ 30 for EA and LA mice (Supplemental Fig. 1). Figure 2 shows that the retained parameters are all clustered closely together, however the excluded parameters show a wide range of variability. Specifically, seven ECG parameters (CV, HRV, pNN5, rMSSD, mean R-amplitude, mean SR-amplitude and QTc dispersion) exceeded the variability criteria in both sexes (male and female) and ages (EA and LA) and were excluded from further analysis (Fig. 2). The variability threshold was exceeded least for QTc dispersion in EA and mean R- and SR-amplitude for LA, however, for the remaining parameters that were excluded, variability was in excess of 2–7 times the threshold.

A PQ-interval reference range is provided for conscious EA and LA mice (Supplemental Fig. 2) however, PQ-interval was excluded from further analysis in this study because data points were only captured in EA and LA mice from one of the ten data-contributing centers. The remaining seven ECG parameters [heart rate (HR), RR-, PR-, ST- and QT-interval, QRS complex, and QT corrected (QTc) using the Mitchell formula (Mitchell et al. 1998)] consistently presented with low variability across the whole IMPC dataset thereby giving high confidence to establish robust, generalizable reference ranges for EA and LA populations on the C57BL/6N-inbred genetic background.

Despite the exclusion of several parameters, the electrical conduction phases of a cardiac cycle were entirely captured by the robust parameters included herein (Fig. 1). The lengths of PR-interval and QRS complex covered the atrial and ventricular depolarization phases (e.g., contraction), whereas lengths of QT- and ST-intervals implied the ventricular repolarization (e.g. relaxation) in voltage over time.

### Assessment of data distribution

**Fig. 3:**
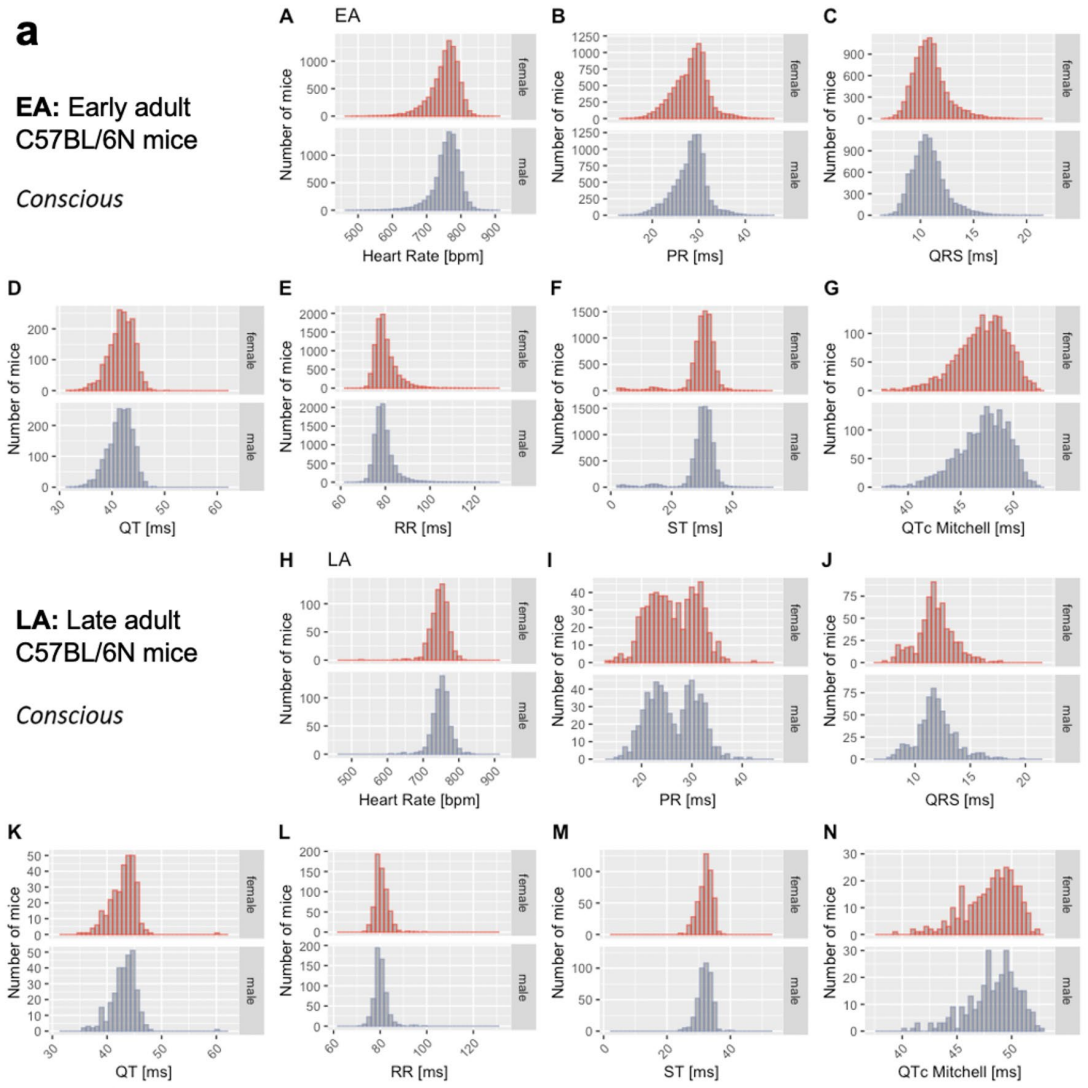

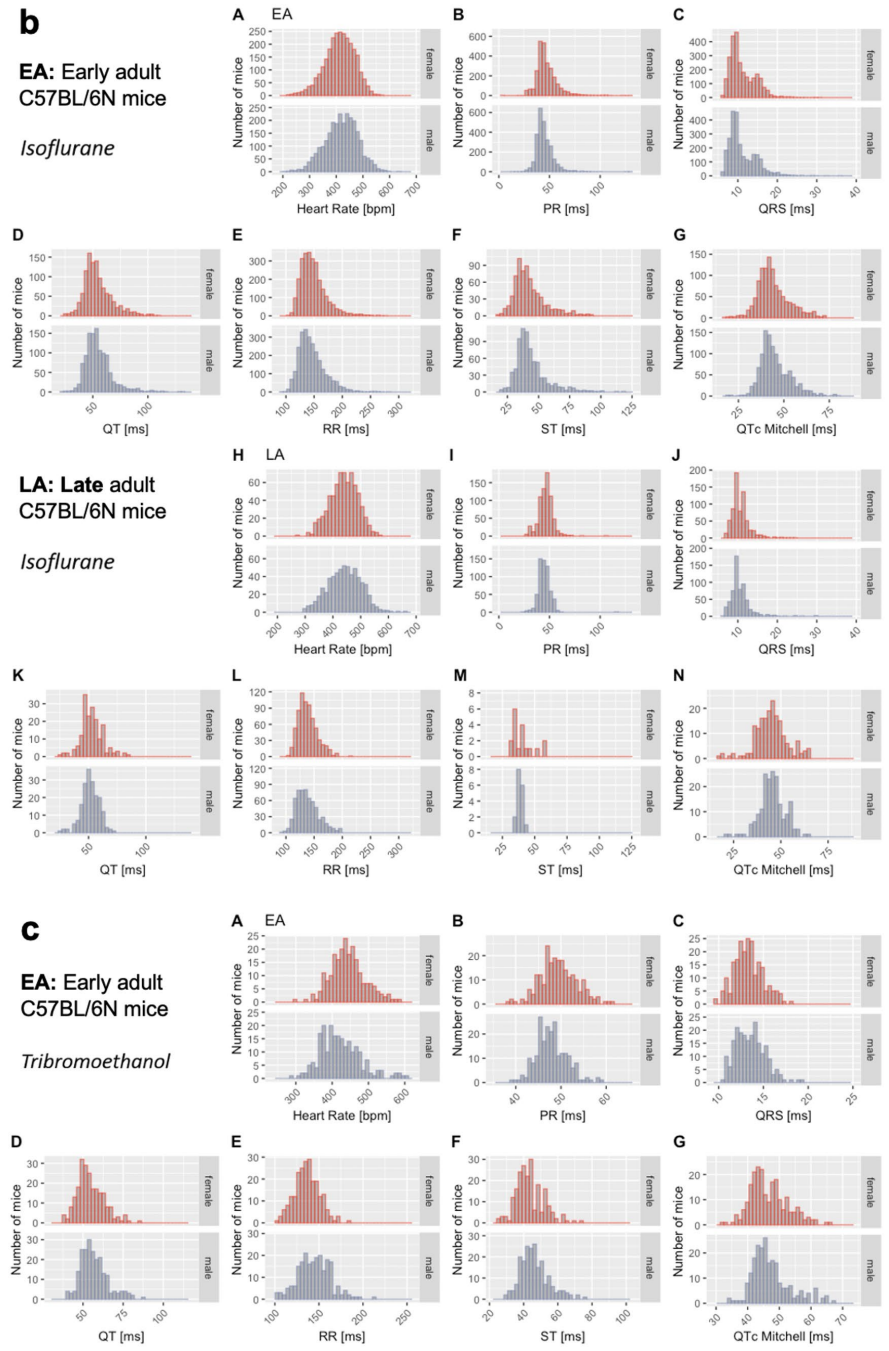
Histograms presenting the distribution of each selected ECG parameter for male and female mice separately. By visual inspection, no sexual dimorphism was apparent. Panel a: Recorded in the conscious state in EA (Subpanels A–G) and LA mice (Subpanels H–N). Panel b: Recorded under isoflurane anesthesia in EA (Subpanels A–G) and LA mice (Subpanels H–N). Panel c: Recorded under tribromoethanol anesthesia in EA (Subpanels A–G). No LA data are available for tribromoethanol anesthesia

The distribution of data were assessed via histograms for the seven selected ECG parameters stratified by sex, age, and anesthetic regime (Fig. 3). This visual representation of the frequency of occurrence per value in the data was useful for revealing conformity to- and deviations from- a normal distribution, for each parameter. Visual inspection of the histograms showed that the data appeared practically normal for parameters PR, QT and QTc Mitchell, and modestly skewed for HR, QRS, ST and RR. To assess normality mathematically, we applied the Shapiro–Wilk test which revealed statistically significant deviation from a normal distribution for some, but not all, ECG parameters. Table 2 presents data as median and 95% reference range (2.5th and 97.5th percentile) to account for the lack of normal distribution of some parameters and to provide a consistent data presentation (Leys et al. 2013). For the sake of completeness, mean, standard deviation and sample size are provided for the seven selected ECG parameters stratified by sex, age, and anesthetic regime in Supplemental Table 2. Interestingly, male, and female data showed similar distributions by visual inspection (Fig. 3). To test the hypothesis that there is no difference between each sex, a simple two-tailed t-test was performed independently for each anesthetic regime and age group, and Cohen’s d was calculated as an effect size measure (Supplemental Figs. 3–5—Panels a and b, stratified by age).

**Table 2:** Median and 95% reference ranges of HR, PR-, QRS complex, QT-, RR- and ST-intervals, and QT corrected (QTc) using the Mitchell formula

Female Conscious	EA	LA
Parameter	Median [95% range]	Median [95% range]
Heart Rate [bpm]	760 [650;815]	748 [700;790]
PR [ms]	28.7 [20.5;35.8]	26.3 [18.2;35]
QRS [ms]	10.7 [8.4;14.8]	11.8 [8.3;15.3]
QT [ms]	42 [36.4;45.8]	43.3 [37.9;46.2]
RR [ms]	79 [73.7;92.9]	80.2 [76;86.1]
ST [ms]	30.8 [13.1;35.8]	32.3 [27;35.3]
QTc Mitchell [ms]	47.4 [41.7;51.1]	48.5 [42.5;51.5]

Female Isoflurane	EA	LA
Parameter	Median [95% range]	Median [95% range]
Heart Rate [bpm]	415.8 [292.6;511.7]	440.5 [339.6;530.3]
PR [ms]	44.5 [29.6;72.7]	46.7 [30.5;58.9]
QRS [ms]	10.1 [7;17.9]	10.1 [7.8;16.6]
QT [ms]	52.1 [35.4;86.9]	52 [29.5;74.2]
RR [ms]	144.4 [117.3;205]	136.2 [113.2;176.7]
ST [ms]	40 [24.3;78]	38.5 [31.9;58.3]
QTc Mitchell [ms]	42.4 [29.9;65.5]	45 [25;62.8]

Female Tribromoethanol	EA	LA
Parameter	Median [95% range]	Median [95% range]
Heart Rate [bpm]	440 [355.8;550.5]	N/A
PR [ms]	49.1 [41.4;57.7]	N/A
QRS [ms]	13.2 [10.6;17]	N/A
QT [ms]	53.5 [40.7;75.7]	N/A
RR [ms]	136.2 [108.9;166.1]	N/A
ST [ms]	42.8 [28.3;63.1]	N/A
QTc Mitchell [ms]	45.2 [36.4;60]	N/A

Male Conscious	EA	LA
Parameter	Median [95% range]	Median [95% range]
Heart Rate [bpm]	764 [661;819.7]	751 [702.3;796.3]
PR [ms]	28.6 [20.4;35]	25.8 [18.2;34.8]
QRS [ms]	10.6 [8.3;14.6]	11.8 [8.6;16.1]
QT [ms]	41.8 [36.6;45.5]	43.3 [38;46.6]
RR [ms]	78.6 [73.2;91.4]	79.9 [75.4;86.1]
ST [ms]	30.6 [12.6;35.6]	32.1 [27.6;35.3]
QTc Mitchell [ms]	47.5 [41.9;51]	48.7 [42.8;51.8]

Male Isoflurane	EA	LA
Parameter	Median [95% range]	Median [95% range]
Heart Rate [bpm]	422.6 [298;534.9]	443.1 [325.1;553.7]
PR [ms]	43.2 [32.5;63.6]	44.3 [34;54.9]
QRS [ms]	10 [7;19.7]	10 [7;17.3]
QT [ms]	52.8 [37.9;89.2]	51.2 [34.6;64.8]
RR [ms]	142 [112.2;201.6]	135.4 [108.4;184.6]
ST [ms]	40.7 [26.9;79.3]	38.7 [35.8;41.9]
QTc Mitchell [ms]	43.4 [32.6;64.9]	45.4 [32.7;58]

Male Tribromoethanol	EA	LA
Parameter	Median [95% range]	Median [95% range]
Heart Rate [bpm]	414.1 [329.9;573.1]	N/A
PR [ms]	47.7 [41.9;55.7]	N/A
QRS [ms]	13.4 [10.8;16.8]	N/A
QT [ms]	55.2 [42.6;76.6]	N/A
RR [ms]	145 [104.7;181.7]	N/A
ST [ms]	45.2 [32.4;65.9]	N/A
QTc Mitchell [ms]	46 [39.3;62.8]	N/A

Data are stratified by sex, age (EA and LA) and conscious state.

Note, there were no data for LA mice anesthetized using tribromoethanol

For some parameters, *p*-values reached significance < 0.001, for others we found no evidence of a difference. However, for all parameters the corresponding Cohen’s *d* value revealed small to negligible effect sizes. We therefore considered the possibility that the large group sizes could be overstating the biological differences between the sexes for some parameters.

To determine the most likely outcome for typical experimental sample sizes we applied a bootstrap analysis stratified by age group. In brief, random sampling (1000 × randomized) of different subsample sizes, ranging from 5 to 100 mice, were applied to test the robustness of the effect for each parameter comparing females and males. The subsample group sizes were chosen to more closely approximate standard experimental groups. The proportion of significant *t*-tests (*p* < 0.05), from the 1000 comparisons, indicates the power to find the sex difference, for that subsample size. If the proportion of significant tests remains near 5% regardless of subsample size, then this indicates the influence of the Type 1, i.e., false positive, error and it is unlikely that experimental group sizes will show a significant effect for this parameter.

Recordings from both conscious (Supplemental Fig. 3) and isoflurane anesthetized mice (Supplemental Fig. 4) show that the ECG parameters consistently have very low proportions of significant tests for sexual dimorphism, with most parameters fluctuating around 5% of tests. Therefore, baseline ECG parameters can be considered likely to be similar in females and males with no sex effect for most experimental purposes.

Tribromoethanol anesthesia (Supplemental Fig. 5) however, reveals weak sexual dimorphism for a subset of parameters. This may be due to a bias from drawing bootstrap samples from a much smaller population than the other conditions, but we cannot exclude the possibility that this anesthetic has a small but significant impact on the sexes.

### Effect of anesthetic agent

**Fig. 4:**
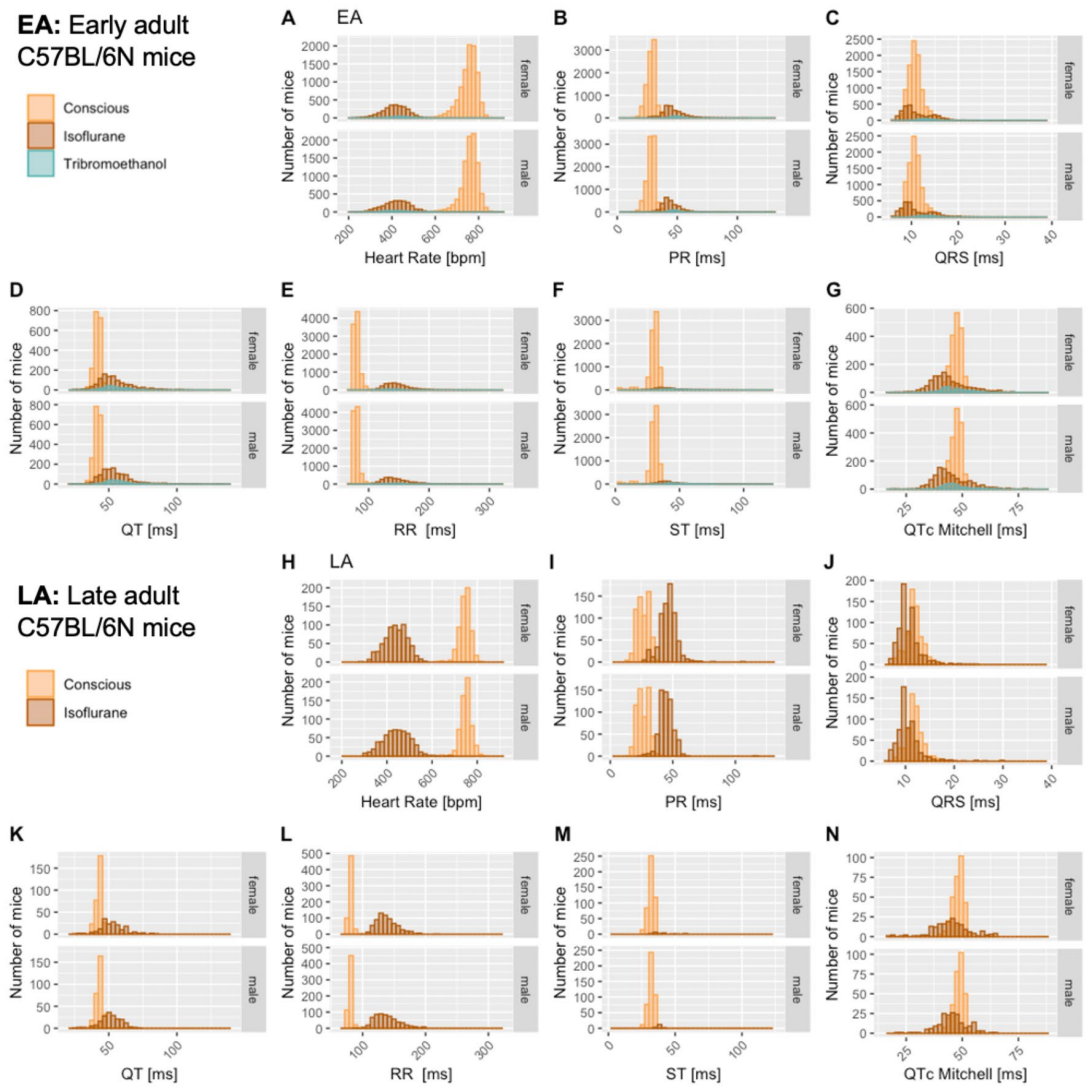
Comparison of the anesthetic regimes with the conscious state recordings. Distribution of the seven selected ECG parameters presented by histograms, stratified for female and male mice in EA (Subpanels A–G) and LA populations (Subpanels H and N). Color code:  Conscious,  Isoflurane and  Tribromoethanol anesthesia

To investigate the effect of different anesthetic agents on cardiac conduction function and ECG profiles, conscious data stratified by sex and age are displayed for comparison with those of isoflurane or tribromoethanol data (Fig. 4). Female data are placed directly above male for ease of visualization. Figure 4 shows distinct distribution clusters for conscious, isoflurane and tribromoethanol groups split by EA (Fig. 4 – Panels A–G) and LA (Fig. 4 – Panels H–N). As before, no data were available for tribromoethanol anesthesia in LA mice.

As expected, the physiological benchmark of highest heart rate in conscious mice compared to anesthetized animals was observed (Fig. 4 – Panels A and H). To assess the differences between EA anesthetic states, we tested conscious versus isoflurane and conscious versus tribromoethanol groups, by a one-way ANOVA with planned comparisons, and observed highly significant differences between those groups (Table 3). Although, for anesthetized mice, some subtle differences in S marker placement were observed across IMPC contributing centers, the consequence of these marker placement differences was overshadowed by the well-established intra-center variability arising from mouse to mouse and day to day data collection (Corrigan et al. 2020; Kafkafi et al. 2005). However, T marker placement in ECG data from conscious and anesthetized mice was substantively different and contributes to the differences in interval duration reported (Table 3). These data clearly show differences in ECG parameters that can be attributed to the anesthetic regime; therefore, it is essential to establish reference ranges separately by condition (conscious or anesthetized) and by anesthetic (isoflurane or tribromoethanol).

**Table 3:** Significant differences between the statistical comparison of conscious versus isoflurane (*p* < .001) and conscious versus tribromoethanol (*p* < .001 to *p* = .004) in female and male mice for HR, PR-, QRS complex, QT-, RR-, ST-interval and QTc Mitchell

FEMALE	Conscious vs Isoflurane	Conscious vs Tribromoethanol
Heart Rate [bpm]	F(1)=120674.4, p<.001	F(1)=2122.7, *p*<.001
PR [ms]	F(1)=21279, *p*<.001	F(1)=872.8, *p*<.001
QRS [ms]	F(1)=78.1, *p*<.001	F(1)=273.2, *p*<.001
QT [ms]	F(1)=1754.3, *p*<.001	F(1)=135, *p*<.001
RR [ms]	F(1)=72292.8, *p*<.001	F(1)=839, *p*<.001
ST [ms]	F(1)=3278.4, *p*<.001	F(1)=285.3, *p*<.001
QTc Mitchell [ms]	F(1)=221, *p*<.001	F(1)=8.3, *p*=.004

MALE	Conscious vs Isoflurane	Conscious vs Tribromoethanol
Heart Rate [bpm]	F(1)=116569.4, *p*<.001	F(1)=2865.6, *p*<.001
PR [ms]	F(1)=23952.9, *p*<.001	F(1)=1142.3, *p*<.001
QRS [ms]	F(1)=71.6, *p*<.001	F(1)=294.8, *p*<.001
QT [ms]	F(1)=2067.7, *p*<.001	F(1)=217.7, *p*<.001
RR [ms]	F(1)=64552.1, *p*<.001	F(1)=1468.3, *p*<.001
ST [ms]	F(1)=4149.9, *p*<.001	F(1)=424.1, *p*<.001
QTc Mitchell [ms]	F(1)=88.9, *p*<.001	F(1)=8.3, p=.004

Test: *p*-value and *F*-value of one-way ANOVA with planned comparison

### Effect of age on ECG parameters

**Fig. 5:**
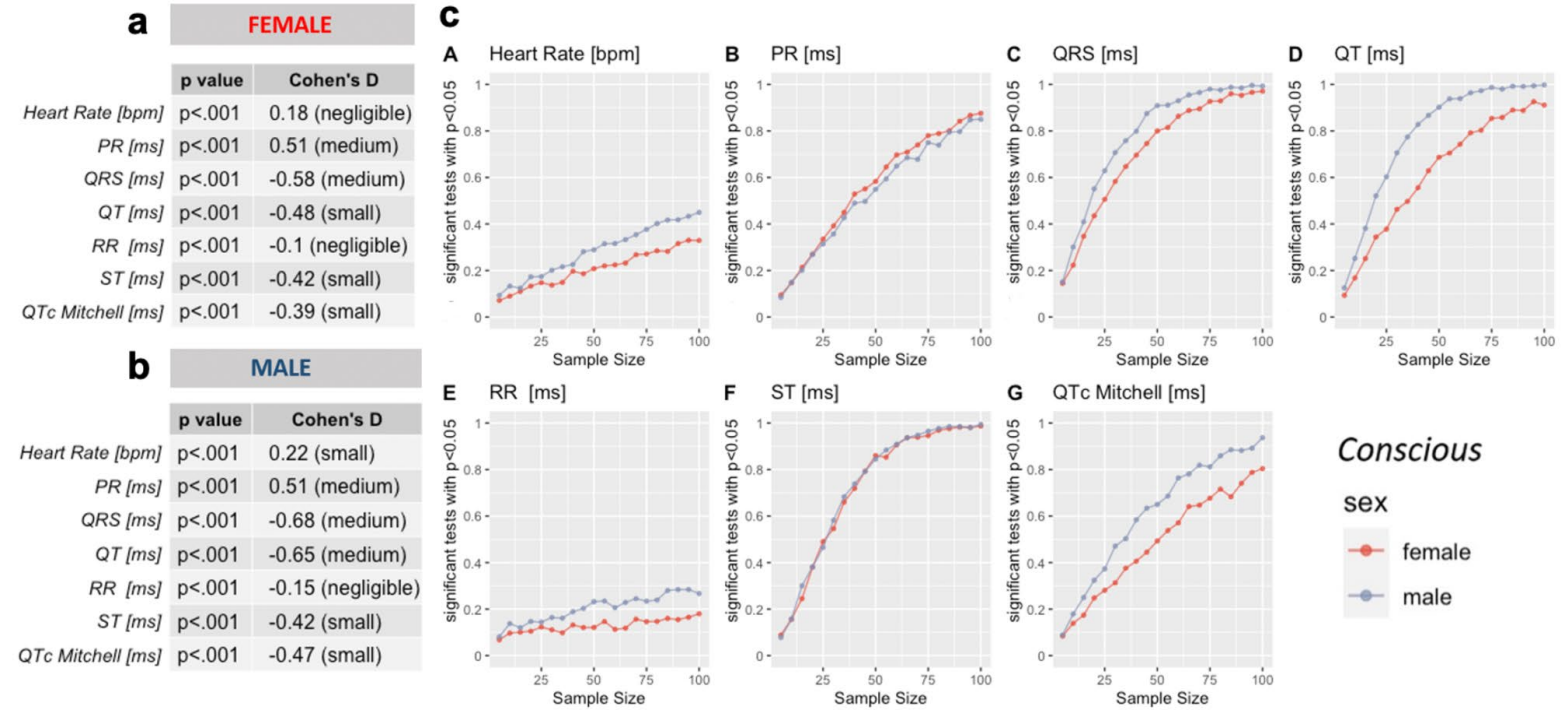
Testing age-differences in conscious mice. T-test results when comparing conscious EA versus LA data show high significance for all parameters (*p* < .001) and negligible to medium Cohen´s *d* standardized effect sizes. Panel a: Females, Panel b: Males. Panel c. Bootstrap analysis of power estimates for sample sizes ranging from 5 to 100 mice, presented for each of the seven selected ECG parameters. Note: X-axis: bootstrapped sample sizes from 5 to 100; Y-axis: proportion of significant tests with *p* < .05

Two different age groups, i.e., mean of 12-weeks (minimum 8 and maximum 16 weeks) old EA and mean of 62 weeks (minimum 52 and maximum 78 weeks) old LA, have made it possible to explore the effect of age on ECG parameters in conscious and isoflurane anesthetized mice. A two-tailed *t*-test was applied to test the difference between the means of EA and LA results in conscious mice (Fig. 5—Panels a and b). *p*-values < 0.001 were reached for all parameters, indicating high statistical significance and the corresponding Cohen’s *d* effect size revealed negligible to medium standardized effect sizes (Fig. 5 – Panels a and b). These strong significance values with comparatively small effect sizes suggest that the unbalanced group sizes influenced the results.

To test the influence of unbalanced group sizes (i.e., large number of EA and smaller number of LA datasets), we applied a bootstrap analysis, this time stratified by sex (Fig. 5 – Panel c). The proportion of significant *t*-tests (*p* < 0.05), from the 1000 comparisons, indicates the power to find the age difference, for that subsample size. This bootstrap analysis demonstrated that parameters with even small to medium effect sizes required relatively large experimental group sizes to attain a conventional > 80% value for power estimates (Cohen 1992; Festing and Altman 2002), e.g. QRS and ST in conscious conditions required a group size of 50 mice to achieve > 80% power with a *p* < 0.05 (Fig. 5 – Panel c, QRS (subpanel C) and ST (subpanel F)). As expected, for parameters with negligible Cohen’s *d* effect sizes, such as HR and RR, increases in sample size do not appreciably increase power (Fig. 5 – Panel c, HR (subpanel A) and RR (subpanel E)). Parameters with less than 80% power even with up to *n* = 100 animals, can be considered likely to be similar in EA and LA with no aging effect for most experimental purposes. Supplemental Fig. 6 presents the equivalent *t*-test, Cohen’s *d* and bootstrap analysis in EA and LA mice anesthetized with isoflurane.

**Fig. 6:**
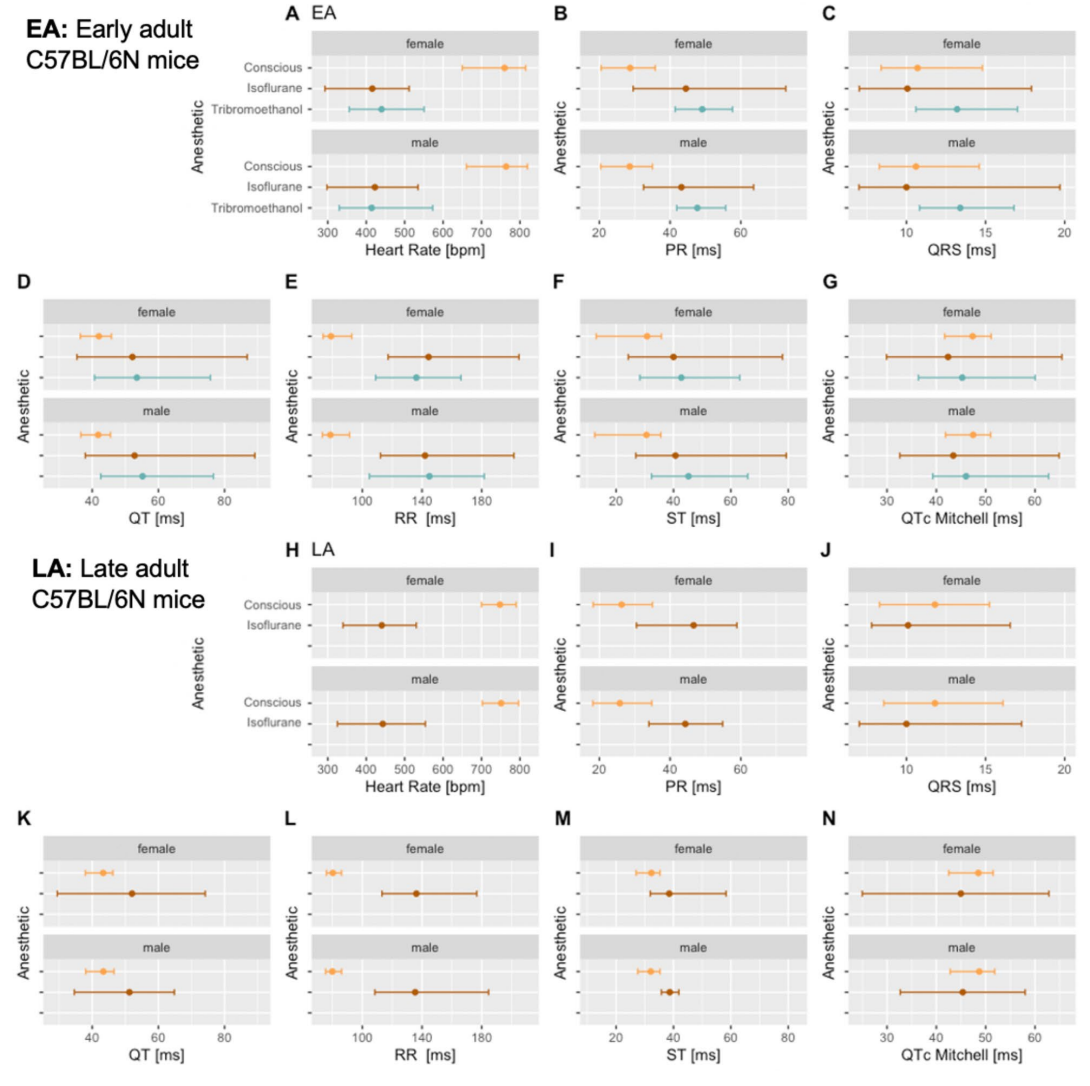
Reference ranges split by anesthetic regimen showing median, and 95% reference ranges (2.5th and 97.5th percentile). Female data are directly above the male data for EA (Subpanels A–G) and LA populations (Subpanels H–N). For the ST-interval in anesthetized mice (Subpanels F and M) data were provided from only one center and for the LA range (Subpanel M) the number of mice was below the recommended number to generate a reliable estimate (Supplementary Table 2). No LA data were available for tribromoethanol anesthesia. Color code:  Conscious,  Isoflurane and  Tribromoethanol anesthesia

In summary, Fig. 6 is a graphical representation of the median and 95% reference ranges (2.5th and 97.5th percentile) broken down by anesthetic regimen with the female data placed directly above equivalent male data for easy visual interpretation, corresponding numeric values are presented in Table 2. This graphical representation clearly shows that anesthetic state strongly influences the reference values of the seven parameters**.**

### Validation of reference ranges using non-IMPC data

**Fig. 7:**
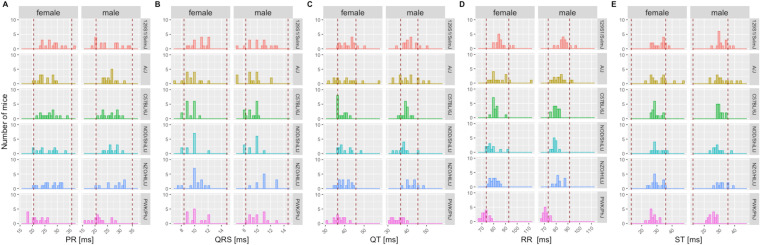
Independent, non-IMPC study on six of the founder strain mice reported in “The Collaborative Cross: A Recombinant Inbred Mouse Population for the Systems Genetic Era” (Threadgill et al. 2011) study, including 129S1/SvlmJ, A/J, C57BL/6 J, NOD/ShiLtJ, NZO/HlLtJ, and PWK/PhJ inbred strains show a close alignment to the reference ranges reported herein for PR-, QRS complex, QT-, RR-, and ST-interval based on multiple C57BL/6N substrains indicating good utility for those reference ranges. Data for HR was not available in this study. Mice were conscious, split by sex and ~ 12 weeks of age, equivalent to the IMPC EA timepoint. Red dotted lines depict the boundaries of the sex-specific reference ranges calculated herein, for each parameter

Mice characterized by the IMPC are all substrains of one commonly used inbred genetic background, C57BL/6N. To test the validity of the reference ranges reported herein beyond C57BL/6N-inbred mice, we used representative control animals from publicly available ECG data including: six founder strains from a collaborative cross study (Threadgill et al. 2011); the Jaxwest1 project (https://phenome.jax.org/projects/Jaxwest1) with seven inbred strains of mice; and the Xing1: Aging study (https://phenome.jax.org/projects/Xing1) (Xing et al. 2009) with 29 inbred strains of which we have included herein the 26 strains with complete ECG data. An additional dataset was included using inbred, wildtype control animals from non-IMPC studies conducted at the German Mouse Clinic where data are available upon request. Validation was also carried out for LA population using 12- and 20-month age groups of the Xing1 study. In each non-IMPC study, where suitable we presented the data split by sex and overlaid with the sex-specific 95% reference range calculated herein for conscious mice. Due to the small sample sizes in a subset of these comparator studies, however, the combined reference ranges for females and males are summarized in Supplemental Table 3 for further comparison. Figure 7 shows the founder strain data from the collaborative cross study overlaid with the reference ranges split by sex whereas Supplemental Fig. 7 illustrates data from the German Mouse Clinic, Supplemental Fig. 8 from the Jaxwest1 and Supplemental Figs. 9–13 depict LA data from the Xing1 study. Of note, HR is not presented throughout as it was not accessible for those studies yet it is indirectly visualized in the RR-interval plot due to the inverse correlation between HR and RR (Kazmi et al. 2016).

Remarkably, and true for all ECG parameters, most non-C57BL/6N values lay within our reference values. There is a subset of outliers that fall outside of the reference ranges which is to be expected with heterogeneity of small size and phenotypic differences seen between inbred mouse strains, particularly wild-derived strains.

## Discussion

Reference ranges for the assessment of abnormal electrocardiograms and cardiac conduction disorders in patients have long been established and are regularly adopted by expert bodies, such as the North American Society of Pacing and Electrophysiology (GURA et al. 2003) and the European Society of Cardiology (Blomström-Lundqvist et al. 2003; Camm et al. 2010). For mouse models, however, there are no such reference ranges.

In this multicenter study, we have established reference ranges using an exceptionally large ECG dataset comprising more than 26,000 wildtype control mice from the International Mouse Phenotyping Consortium (IMPC). The goal of the IMPC is to extend the functional annotation of the mammalian genome via the large-scale production and phenotypic characterization of single gene knockout mouse strains for all protein-coding genes. The phenotypic pipeline used to characterize these knockout strains included cardiac electrophysiology assessment using ECG. For each knockout strain characterized, we also assessed wildtype control animals matched for age, sex and genetic background. The ECG data from these C57BL/6N wildtype control mice hold extraordinary value and represent the focus of the current study.

Thus, this study represents a large mouse data set and allows the crucial understanding of the effects of sex, age, and anesthesia on electrocardiograms in mice. To this end, we introduced a stepwise refinement of the data analysis and started with an in-depth assessment of the variability of 15 ECG parameters gathered in the IMPC. We identified seven clinically relevant ECG parameters that were highly robust and had low variability. We excluded the remaining eight ECG parameters because of the excessive level of inter-mouse variability they displayed. Five of the eight excluded parameters were direct measures of heart rate variability (HRV), or represented parameters derived from HRV (HRV, pNN5, rMSSD, mean R-amplitude and mean SR-amplitude). HRV depicts the change in the time interval between successive heartbeats and is an index of the parasympathetic nervous system (Sassi et al. 2015; Singh et al. 2018). HRV measurement is very sensitive to experimental methods (e.g. acclimation time, ECG sampling rate, and duration of recording), and has been shown to be incompatible with a high-throughput data collection setup such as that used by the IMPC (Electrophysiology 1996; Sammito and Böckelmann 2016). Next, CV provides an indication of the function of the parasympathetic nerve and the autonomic nervous system through the physiological phenomenon of RR variation (Saito et al. 2007). Such measurements, however, require stable and prolonged measurement times to be meaningful, which, as stated above for HRV, we do not have in the context of the high-throughput testing paradigm used herein. Similarly, this susceptibility to broad variability in short duration measurements also applies to the parameter QT dispersion, which is defined as the difference between the longest and shortest QT-interval in one of the surface ECG leads and quantifies the spatial inhomogeneity of ventricular repolarization. Mainly for methodological reasons, parameters with high variability were excluded here, but PR-interval is the exception. This parameter was only collected by one center and therefore not included in the overall evaluation, but the values were made available in full in the supplemental materials. Despite the exclusion of those parameters, the robust ECG parameters that were included entirely captured the electrical conduction phases of a cardiac cycle and provided a comprehensive ECG evaluation.

Understanding the sex-related impact on ECG is crucial for ensuring robust reference values. In this study, we were able to show that the values for HR, RR-, PR-, ST- and QT-interval, QRS complex, and QT corrected (QTc) using the Mitchell formula (Mitchell et al. 1998) are comparable in female and male mice with negligible sexual dimorphism. There may, however, be small sex differences for some parameters depending on the anesthetic agent. This observation is of key importance, and in part consistent with previous mouse data (Karp et al. 2017). Whilst sexual dimorphism was not overtly apparent in inbred mice in the absence of any environmental, pharmacological or genetic perturbations, the literature clearly supports sex differences in heart health (Pak et al. 2021) and therefore our recommendation is that both sexes are included in any experimental design assuming that post-treatment we may detect sex differences.

Anesthetics cause a dose-dependent decrease in myocardial contractile force and associated ECG alterations with the most familiar landmark of decreased HR (Edrich et al. 2008). Our observations are that presence of anesthesia matters, we confirm a decreased heart rate in anesthetized mice and go on to reveal distinctions in isoflurane inhalation anesthesia and intraperitoneal injected tribromoethanol-induced anesthesia (Chu et al. 2006; Shintaku et al. 2014). These distinctions are pivotal and to emphasize them we mapped the effects of three different states (conscious, isoflurane and tribromoethanol anesthesia) on seven ECG parameters in detail and present anesthesia-specific reference values.

HR is an important determinant of cardiovascular performance defined by the activity of the sinoatrial node, the so-called pacemaker of the heart. The dysfunction of the sinoatrial node increases with age, and HR decreases due to tissue, cellular, and molecular mechanisms that underlie the reduction in pacemaker activity with age (Alings et al. 1995; Moghtadaei et al. 2016; Peters et al. 2020). Interestingly, we did not observe any strong age-related ECG changes in the absence of any pharmacological, environmental, or genetic challenges in inbred C57BL/6N mice. The differences in the reference ranges of 12-week-old mice compared to 62-week-old mice were negligible. Our step-by-step analysis of these data using bootstrapping showed that age-related ECG effects are more likely, if at all, to be detected using large group sizes (*n* > 50). This dependency on the group size can be used as a guide for experimental design when considering aging. It is possible that studying a population older than 62 weeks of age would have identified larger age-related changes in ECG parameters.

In the IMPC, we control for genetic diversity using C57BL/6N-inbred background substrains thereby focusing our comparison on the genetic perturbation of interest i.e., the single gene that is knocked out on this common genetic background. The transferability from the C57BL/6N background used here, however, was demonstrated by independently validating the ranges using data from a broad spectrum of non-IMPC C57BL/6N and C57BL/6 J mice, and other inbred and wild-derived inbred strains. This validation indicates that C57BL/6N-based reference values represent a robust and comprehensive indicator of normality for many strains and can be used as a starting point for experimental investigations of cardiac function in the mouse. A subset of outlier strain-parameter combinations were identified, for example, the RR-interval in PWK/PhJ mice fell below the C57BL/6N-based reference range reported herein. The particularly small body weight of this wild-derived genetically diverse strain (Bonhomme et al. 1984; Kollmus et al. 2020; von Deimling et al. 1988) is consistent with increased HR and therefore explains their decreased RR-interval.

Each study has its limitations. *P*-wave interval alone was not reported here, however the reported PR-interval did allow discrimination of atrioventricular conduction time (Clark and Prystowsky 2021). In addition, the PQ-interval was only recorded at one contributing center and exclusively in conscious mice, yet this large sample size (*n* = 11,538 EA mice) that was equally distributed for sex, yielded a valuable PQ reference range that is provided in full in Supplemental Fig2. The majority of data included in this study were collected on conscious mice using the non-invasive, ECGenie methodology. Given the large sample size collected, this approach represented a huge 3Rs benefit (Hubrecht and Carter 2019; Tannenbaum and Bennett 2015). However, the relatively low-resolution of the ECGenie technique meant that annotation of the J wave, a commonly recognized feature of the mouse ECG, was omitted from this study. The difference in T marker positioning between ECG data from conscious and anesthetized mice was substantive and contributed to the statistically significant differences in time intervals involving T (QT, QTc and ST). However, we are unable to decouple the contribution of the anesthetic agent and the T marker location. Taken together these differences highlight the urgent need for a standardized and agreed annotation schema that accommodates the variable sensitivity of ECG recording methods.

The limiting factors for the tribromoethanol reference range data are that it was generated for 12-week-old mice only and the group size was the smallest of all conditions reported herein [446 mice distributed equally between sex (*n* = 226 female; *n* = 220 male)]. However, Solberg and colleagues (Solberg 1983) report that for a reliable estimate, a minimum of 120 values should be included for any reference range calculation. The sample size we used for tribromoethanol far exceeds this minimum and should therefore yield a representative range. The reference ranges are limited to the techniques and anesthetics described and are not intended for other ECG methodologies, such as cardiovascular telemetry, or other anesthetic agents, such as ketamine.

The reference ranges reported herein can be used to demarcate typical values for an experimental control group of mice on a C57BL/6N genetic background, for a given sex and age. They are not a substitute for contemporaneous control groups in any experimental design, but they indicate the likely values of that control group, thereby acting as a quality assurance tool. These reference ranges provide the information necessary to assess the changes in ECG parameters resulting from pharmacological, environmental, or genetic perturbations for experiments conducted on the commonly used C57BL/6N genetic background.

In summary, we have created a unique and comprehensive map of ECG reference ranges that will be foundational for future mouse studies. While based on inbred mouse substrains that are C57BL/6N in origin, these reference ranges have utility across different mouse strains and are important guides in studies of electrical conductivity disorders.

## Supplementary Information

Below is the link to the electronic supplementary material.

Supplementary file1 (PDF 19886 KB)—Supplemental Table 1: Definition and unit of measure for each ECG parameter reported. Supplemental Table 2: Comprehensive overview of mean, standard deviation, and sample number for each of the seven selected ECG parameters stratified by conscious state (conscious, anesthetized with isoflurane, or anesthetized with tribromoethanol), age (EA and LA timepoint) and sex (Panel a. Females; Panel b. Males). Supplemental Table 3: Comprehensive overview of median and 95% reference ranges (2.5th and 97.5th percentile) as well as mean, standard deviation, and sample number for each of the seven selected ECG parameters, stratified by conscious state (conscious, anesthetized with isoflurane, or anesthetized with tribromoethanol) and age (EA and LA timepoint). Sex is combined (females plus males) for this analysis to generate a both-sex-combined reference range. Supplemental Figure 1: Quartile-based CV (QCV), defined as interquartile range (IQR) (75-25%) relative to the median (100*IQR/median), analysis of data split by sex (female and male) and age (EA and LA) identified parameters with excess variability (QCV ≥30% for EA and LA timepoint) that were excluded from further analysis (white bars). pNN5 could not be calculated due to a zero denominator, therefore it was not displayed. Parameters in blue were below the QCV threshold and were retained for further analysis. In the QCV analysis, but not in the COV, there were three parameters (light blue bars) in the LA population (HR, RR and PR) that marginally exceeded the limit of QCV ≥30 but were retained. Supplemental Figure 2: Histograms presenting the distribution of PQ-interval data along with calculated ranges (mean ± SD and median and 95% reference range) for conscious EA and LA mice stratified by sex. These calculations are based on data from one contributing center (German Mouse Clinic). Supplemental Figure 3: Testing sex-differences in conscious mice. T-test results when comparing data from conscious male and female animals for each of the seven selected ECG parameters, stratified by age (EA and LA timepoint) demonstrated that some parameters show high significance (p<.001), while for others there was no indication of sexual dimorphism. Associated Cohen´s *d* standardized effect sizes were negligible. Panel a shows EA timepoint, Panel b shows LA timepoint, and Panel c shows bootstrap analysis of power estimates for female and male samples ranging from 5-100 mice, presented for each of the seven selected ECG parameters. Note: X-axis: bootstrapped sample sizes from 5-100; Y-axis: proportion of significant tests with p<.05. Supplemental Figure 4: Testing sex-differences in isoflurane anesthetized mice. T-test results when comparing data from isoflurane anesthetized male and female animals for each of the seven selected ECG parameters, stratified by age (EA and LA timepoint) demonstrated that some parameters show high significance (p<.001), while for others there was no indication of sexual dimorphism. Associated Cohen´s *d* standardized effect sizes were negligible to small. Panel a. shows EA timepoint. Panel b. shows LA timepoint, and Panel c. shows bootstrap analysis of power estimates for female and male samples ranging from 5-100 mice, presented for each of the seven selected ECG parameters. Note: X-axis: bootstrapped sample sizes from 5-100; Y-axis: proportion of significant tests with p<.05. Supplemental Figure 5: Testing sex-differences in tribromoethanol anesthetized mice. T-test results when comparing data from tribromoethanol anesthetized male and female animals for each of the seven selected ECG parameters, stratified by age (EA but no LA timepoint) demonstrated that some parameters show high significance (p<.001), while for others there was no indication of sexual dimorphism. Associated Cohen´s *d* standardized effect sizes were negligible to small. Panel a. shows EA timepoint, and Panel b. shows bootstrap analysis of power estimates for female and male samples ranging from 5-100 mice, presented for each of the seven selected ECG parameters. Note: X-axis: bootstrapped sample sizes from 5-100; Y-axis: proportion of significant tests with p<.05. Supplemental Figure 6: Testing age-differences in isoflurane anesthetized mice. There is little to no aging effect by equivalent t-test, Cohen’s *d* and bootstrap analysis in EA and LA mice anesthetized with isoflurane. Some parameters show high significance (p<.001), while for others there was no indication of an age effect. Associated Cohen´s *d* standardized effect sizes were negligible to small. Panel a. shows females. Panel b. shows males and Panel c. shows bootstrap analysis of power estimates ranging from 5-100 mice, presented for each of the seven selected ECG parameters. Note: X-axis: bootstrapped sample sizes from 5-100; Y-axis: proportion of significant tests with p<.05. Supplemental Figure 7: Wildtype control animals from three non-IMPC studies tested at the German Mouse Clinic (https://www.mouseclinic.de/) with a standard sample size (20-30 control animals per study) on the background strains C57BL/6J, C57BL/6NJ and FVB show a close alignment to the reference ranges reported herein for HR, PR-, QRS complex, QT-, RR-, and ST-interval, and QTc Mitchell values based on multiple C57BL/6N substrains indicating good utility for those reference ranges. Mice were conscious, split by sex and ~12 weeks of age, equivalent to the IMPC EA timepoint. Red dotted lines depict the boundaries of the sex-specific reference range calculated herein, for each parameter. Supplemental Figure 8: Seven inbred strains of the Jaxwest1 project 129S1/SvImJ, A/J, BALB/cJ, C57BL/6J, DBA/2J, NOD/ShiLtJ and SJL/J show a close alignment to the reference ranges reported herein for PR-, QRS complex, QT-, RR-, and ST-interval based on multiple C57BL/6N substrains indicating good utility for those reference ranges. Mice were conscious, split by sex and ~12 weeks of age, equivalent to the IMPC EA timepoint. Red dotted lines depict the boundaries of the sex-specific reference range calculated herein, for each parameter. Supplemental Figure 9-13: The Xing1 Aging study includes 29 inbred strains of which 26 have been included here: 129S1/SvImJ, A/J, BALB/cByJ, BTBR *T*^*+*^* Itpr3*^*tf*^/J, BUB/BnJ, C3H/HeJ, C57BL/10J, C57BL/6J, C57BLKS/J, C57BR/cdJ, C57L/J, CBA/J, DBA/2J, FVB/NJ, KK/HIJ, LP/J, MRL/MpJ, NOD.B10Sn-*H2*^*b*^/J, NON/ShiLtJ, NZO/HlLtJ, NZW/LacJ, P/J, PL/J, RIIIS/J, SM/J, and SWR/J. The data show a close alignment to the reference ranges reported herein for PR-, QRS complex, QT-, RR-, and ST-interval based on multiple C57BL/6N substrains indicating good utility for those reference ranges. Mice were conscious, split by sex and ~12 months or ~20 months of age, equivalent to the IMPC LA time ranges with a minimum of 52 and a maximum of 78 weeks of age. Red dotted lines depict the sex-specific boundaries of the reference range calculated herein, for each parameter.

## Data Availability

All data used are available to the public for download at the IMPC (https://www.mousephenotype.org/data/previous-releases/15.0).
