# Supplementary material for: Comprehensive ECG reference intervals in C57BL/6N substrains provide a generalizable guide for cardiac electrophysiology studies in mice
Source: Mamm Genome. 2023 Jun 9;34(2):180–99. doi: 10.1007/s00335-023-09995-y (PMC10290602; doi:10.1007/s00335-023-09995-y)

Supplemental Table 1:

Abbreviation	Parameter Definition	Unit	
CV	Coefficient of variation (CV = SD/mean x 100) of the R-R interval	%	percent
HR	Heart rate	bpm	beats per minute
HRV	Heart rate variability	bpm	beats per minute
mean R-amplitude	Average (mean) amplitude of the R wave from all R-waves in the measurement	mV	millivolt
mean SR-amplitude	Average (mean) amplitude of the SR wave from all SR-waves in the measurement	mV	millivolt
pNN5	The fraction of consecutive NN intervals that differ by more than 5 ms	counts	counts
PQ interval	Time from the start of the P-wave to the beginning of Q-wave	ms	milliseconds
PR interval	Time from the start of the P-wave to the peak of the QRS-complex	ms	milliseconds
QRS complex	Duration of the QRS-complex	ms	milliseconds
QT interval	Time from the onset of the Q-wave to the end of the T-wave	ms	milliseconds
QTc	QT corrected for HR (applying the Mitchell formula)	ms	milliseconds
QTc dispersion	Maximum QT-interval minus minimum QT-interval corrected for HR	ms	milliseconds
rMSSD	Root Mean Square of Successive Differences	ms	milliseconds
RR interval	Interval between successive R-peaks	ms	milliseconds
ST interval	End point of S wave to start point of T wave	ms	milliseconds

Supplemental Table 2:

a

	FEMALE					
	Conscious		*Isoflurane*		*Tribromoethanol*	
	EA	LA	EA	LA	EA	
Parameter	mean ± sd (n)	mean ± sd (n)	mean ± sd (n)	mean ± sd (n)	mean ± sd (n)	No LA data available
Heart Rate [bpm]	753 ± 41.6 (9238)	745.8 ± 25.4 (620)	413 ± 55.8 (2670)	439.5 ± 49.5 (693)	442.4 ± 49.4 (226)	
PR [ms]	28.3 ± 3.7 (9240)	26.4 ± 4.8 (620)	46.9 ± 10.5 (2664)	46.2 ± 7.1 (692)	49.4 ± 4.1 (225)	
QRS [ms]	10.9 ± 1.6 (9238)	11.8 ± 1.7 (620)	11.2 ± 3.2 (2672)	10.7 ± 2.2 (693)	13.4 ± 1.8 (217)	
QT [ms]	41.8 ± 2.4 (1840)	43 ± 2.4 (306)	54.5 ± 12.8 (1106)	52.5 ± 10 (179)	54.7 ± 9.4 (225)	
RR [ms]	80.1 ± 5 (9239)	80.6 ± 2.8 (619)	148.4 ± 22.7 (2670)	138.4 ± 16.5 (693)	137.1 ± 16.2 (225)	
ST [ms]	29.9 ± 5.2 (8012)	32 ± 2.2 (468)	42.6 ± 12.8 (713)	40.4 ± 8.2 (18)	43.8 ± 9.1 (209)	
QTc Mitchell [ms]	47.2 ± 2.4 (1839)	48.1 ± 2.4 (305)	43.9 ± 8.6 (1104)	44.9 ± 8.4 (179)	46.7 ± 6.3 (224)	

b

	MALE					
	Conscious		*Isoflurane*		*Tribromoethanol*	
	EA	LA	EA	LA	EA	
Parameter	mean ± sd (n)	mean ± sd (n)	mean ± sd (n)	mean ± sd (n)	mean ± sd (n)	No LA data available
Heart Rate [bpm]	758.9 ± 39.7 (9237)	750.3 ± 24.8 (588)	419.8 ± 61.4 (2598)	443 ± 60.2 (610)	422.7 ± 57.7 (220)	
PR [ms]	28.1 ± 3.5 (9238)	26.3 ± 4.8 (589)	44.8 ± 8.4 (2590)	44.8 ± 6 (600)	48 ± 3.6 (219)	
QRS [ms]	10.8 ± 1.6 (9237)	11.9 ± 1.7 (588)	11.1 ± 3.4 (2598)	10.6 ± 2.5 (610)	13.5 ± 1.7 (214)	
QT [ms]	41.6 ± 2.3 (1825)	43.1 ± 2.4 (296)	55.1 ± 12.7 (1140)	51.4 ± 7.8 (185)	56.7 ± 8.4 (219)	
RR [ms]	79.4 ± 4.8 (9238)	80.1 ± 2.8 (588)	146.3 ± 23.8 (2597)	138.1 ± 19.6 (610)	144.5 ± 18.9 (220)	
ST [ms]	29.8 ± 5.2 (8003)	32 ± 2.1 (439)	44.1 ± 13.4 (741)	38.5 ± 1.9 (17)	46.4 ± 8.3 (206)	
QTc Mitchell [ms]	47.2 ± 2.4 (1825)	48.3 ± 2.3 (295)	45.2 ± 8.3 (1140)	45.5 ± 6.8 (185)	47.3 ± 6.1 (219)	

Supplemental Table 3:

Combined FEMALE and MALE

	Conscious		*Isoflurane*		*Tribromoethanol*	
	**EA**	**LA**	**EA**	**LA**	**EA**	
Parameter	median [95% range]	median [95% range]	median [95% range]	median [95% range]	median [95% range]	No LA data available
Heart Rate [bpm]	762 [653.9;817.6]	750 [700.1;792.9]	419 [295;524.1]	441.4 [335.2;540.3]	430 [334.3;558.2]	
PR [ms]	28.6 [20.4;35.5]	26 [18.2;35]	43.8 [30.7;68.7]	45.6 [31.2;56.7]	48.4 [41.6;57.4]	
QRS [ms]	10.7 [8.3;14.7]	11.8 [8.4;15.7]	10 [7;18.7]	10.1 [7.1;16.8]	13.3 [10.8;17]	
QT [ms]	41.9 [36.5;45.7]	43.3 [37.8;46.4]	52.4 [36.2;87.8]	51.6 [30.3;70.4]	54.3 [41.6;75.8]	
RR [ms]	78.8 [73.4;92.3]	80 [75.7;86.1]	143.3 [114.5;203.3]	135.9 [111.1;179.5]	139.5 [107.4;178.3]	
ST [ms]	30.7 [12.8;35.7]	32.2 [27;35.3]	40.3 [24.8;78.6]	38.7 [32.1;58.3]	43.7 [30.7;64.8]	
QTc Mitchell [ms]	47.4 [41.8;51.1]	48.6 [42.6;51.7]	42.9 [31.1;64.9]	45.3 [28.6;60.8]	45.8 [36.8;61.5]	

	**EA**	**LA**	**EA**	**LA**	**EA**	
Parameter	mean ± sd (n)	mean ± sd (n)	mean ± sd (n)	mean ± sd (n)	mean ± sd (n)	No LA data available
Heart Rate [bpm]	756 ± 40.8 (18475)	748 ± 25.2 (1208)	416.4 ± 58.7 (5268)	441.2 ± 54.8 (1303)	432.7 ± 54.5 (446)	
PR [ms]	28.2 ± 3.6 (18478)	26.4 ± 4.8 (1209)	45.9 ± 9.6 (5254)	45.6 ± 6.6 (1292)	48.7 ± 3.9 (444)	
QRS [ms]	10.8 ± 1.6 (18475)	11.8 ± 1.7 (1208)	11.2 ± 3.3 (5270)	10.7 ± 2.4 (1303)	13.4 ± 1.7 (431)	
QT [ms]	41.7 ± 2.4 (3665)	43 ± 2.4 (602)	54.8 ± 12.7 (2246)	52 ± 9 (364)	55.7 ± 8.9 (444)	
RR [ms]	79.7 ± 4.9 (18477)	80.4 ± 2.8 (1207)	147.4 ± 23.3 (5267)	138.3 ± 18 (1303)	140.8 ± 18 (445)	
ST [ms]	29.9 ± 5.2 (16015)	32 ± 2.2 (907)	43.3 ± 13.2 (1454)	39.5 ± 6 (35)	45.1 ± 8.8 (415)	
QTc Mitchell [ms]	47.2 ± 2.4 (3664)	48.2 ± 2.3 (600)	44.5 ± 8.5 (2244)	45.2 ± 7.6 (364)	47 ± 6.2 (443)	

Supplemental Figure 1:

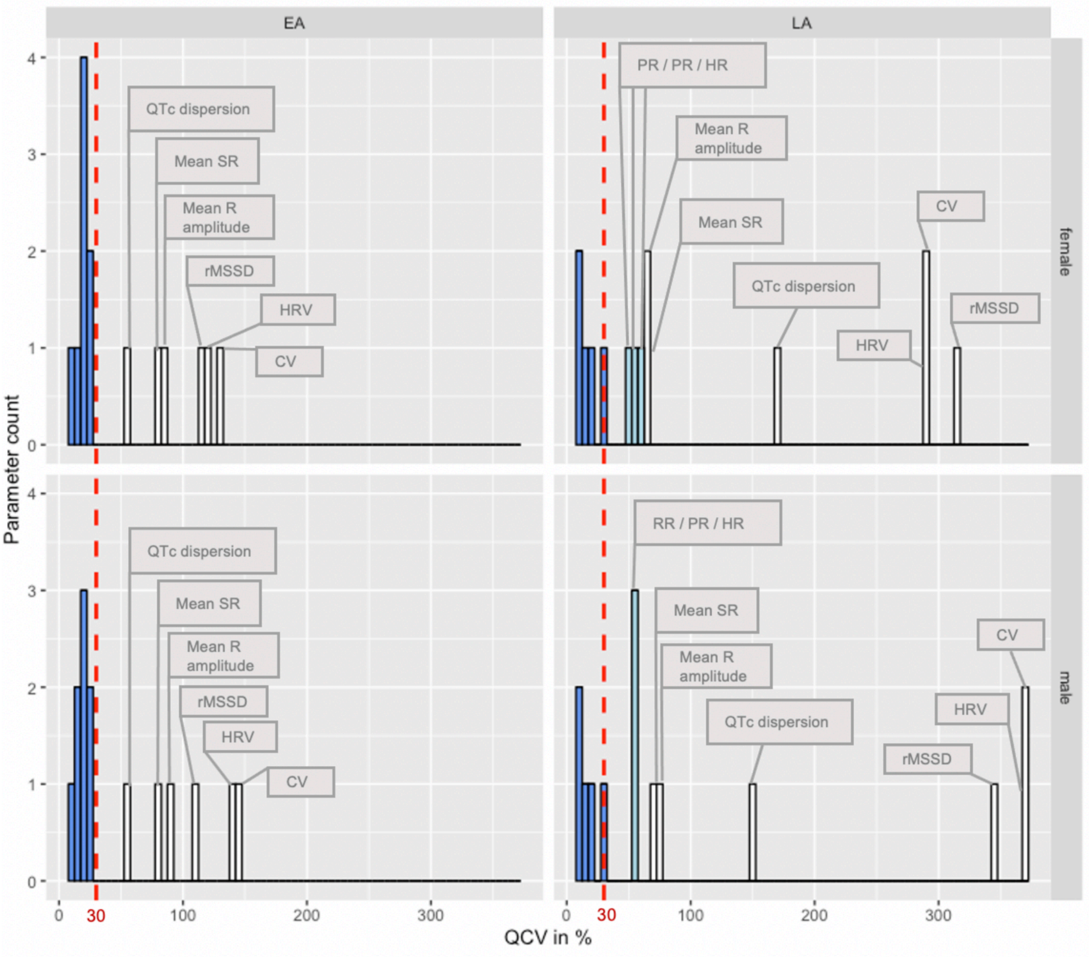

Supplemental Figure 2:

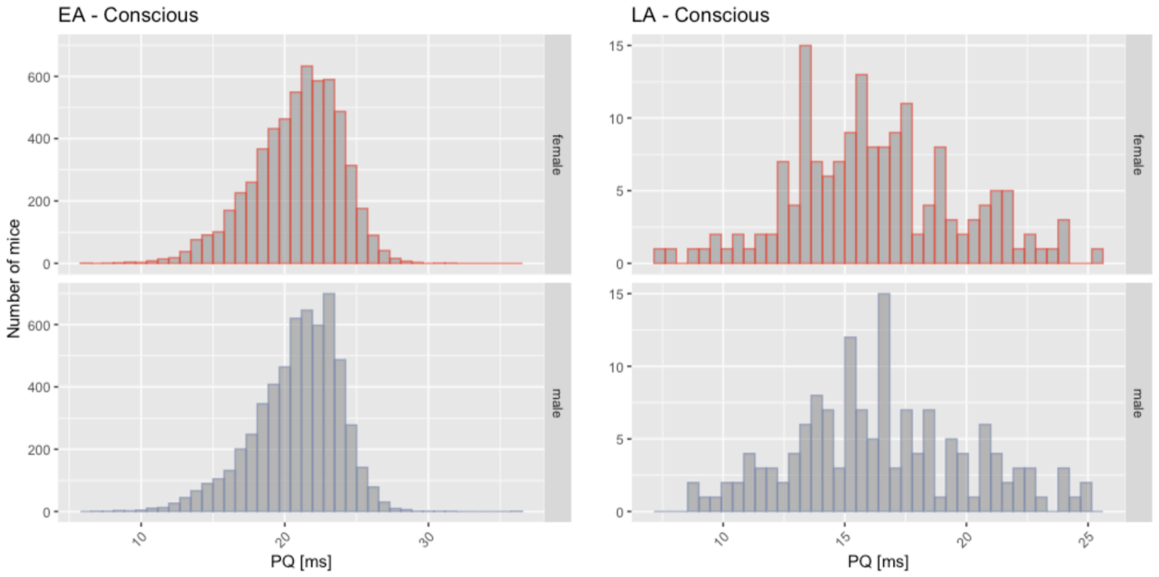

	mean ± sd (n)	median [95% range]
PQ [ms] - female	20.86 ± 3.03 (5769)	21.2 [14.1;25.8]
PQ [ms] - male	20.87 ± 2.97 (5769)	21.3 [13.9;25.7]
PQ [ms] - both	20.87 ± 3 (11538)	21.3 [14;25.8]

mean ± sd (n)	median [95% range]
16.25 ± 3.48 (163)	15.9 [9.6;23.6]
16.51 ± 3.65 (144)	16.2 [10;24.1]
16.37 ± 3.56 (307)	16.1 [9.7;23.9]

Supplemental Figure 3:

a EA

	p value	Cohen's D
Heart Rate [bpm]	p<.001	-0.15 (negligible)
PR [ms]	p=.003	0.04 (negligible)
QRS [ms]	p<.001	0.06 (negligible)
QT [ms]	p=.003	0.1 (negligible)
RR [ms]	p<.001	0.14 (negligible)
ST [ms]	p=.245	0.02 (negligible)
QTc Mitchell [ms]	p=.715	-0.01 (negligible)

b LA

	p value	Cohen's D
Heart Rate [bpm]	p=.002	-0.18 (negligible)
PR [ms]	p=.877	0.01 (negligible)
QRS [ms]	p=.397	-0.05 (negligible)
QT [ms]	p=.534	-0.05 (negligible)
RR [ms]	p=.006	0.16 (negligible)
ST [ms]	p=.586	0.04 (negligible)
QTc Mitchell [ms]	p=.255	-0.09 (negligible)

c

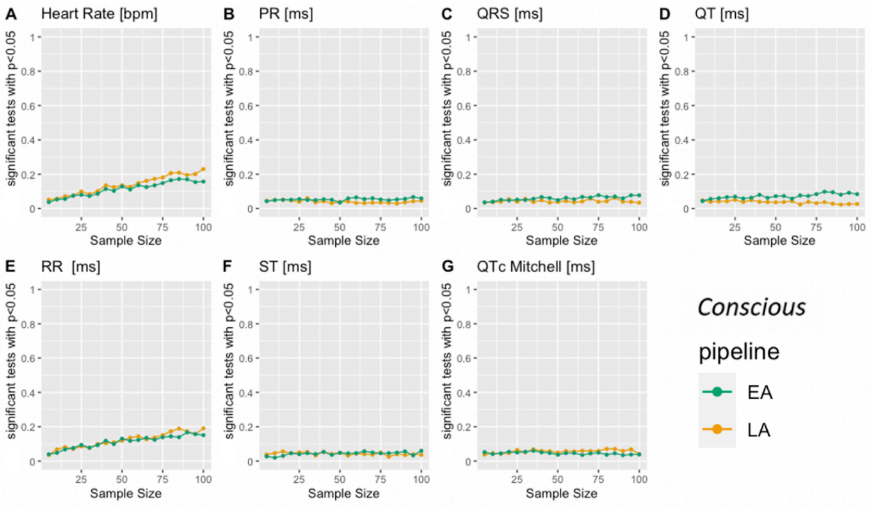

Supplemental Figure 4:

a

EA	p value	Cohen's D
Heart Rate [bpm]	p<.001	-0.12 (negligible)
PR [ms]	p<.001	0.21 (small)
QRS [ms]	p=.288	0.03 (negligible)
QT [ms]	p=.248	-0.05 (negligible)
RR [ms]	p=.002	0.09 (negligible)
ST [ms]	p=.032	-0.11 (negligible)
QTc Mitchell [ms]	p<.001	-0.15 (negligible)

b

LA	p value	Cohen's D
Heart Rate [bpm]	p=.260	-0.06 (negligible)
PR [ms]	p<.001	0.22 (small)
QRS [ms]	p=.394	0.05 (negligible)
QT [ms]	p=.253	0.12 (negligible)
RR [ms]	p=.766	0.02 (negligible)
ST [ms]	p=.347	0.32 (small)
QTc Mitchell [ms]	p=.451	-0.08 (negligible)

c

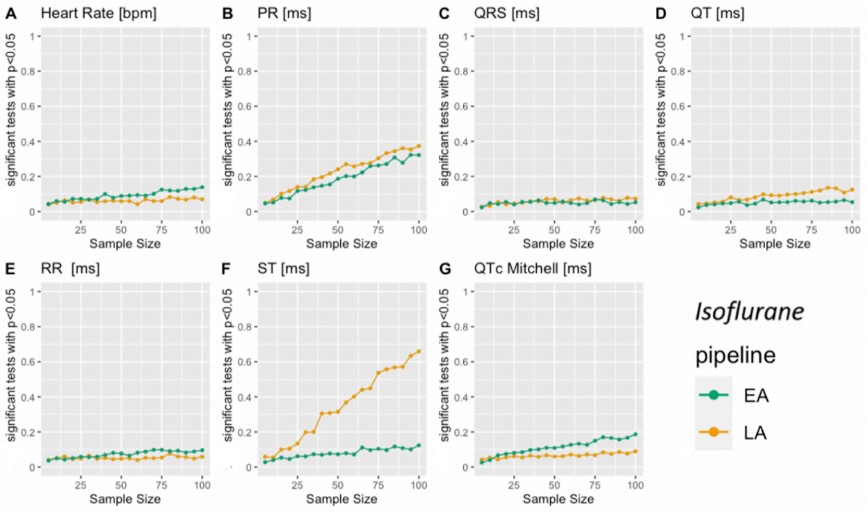

Supplemental Figure 5:

a

EA	p value	Cohen's D
Heart Rate [bpm]	p<.001	0.37 (small)
PR [ms]	p<.001	0.36 (small)
QRS [ms]	p=.522	-0.06 (negligible)
QT [ms]	p=.018	0.22 (small)
RR [ms]	p<.001	-0.42 (small)
ST [ms]	p=.003	0.29 (small)
QTc Mitchell [ms]	p=.283	-0.1 (negligible)

b

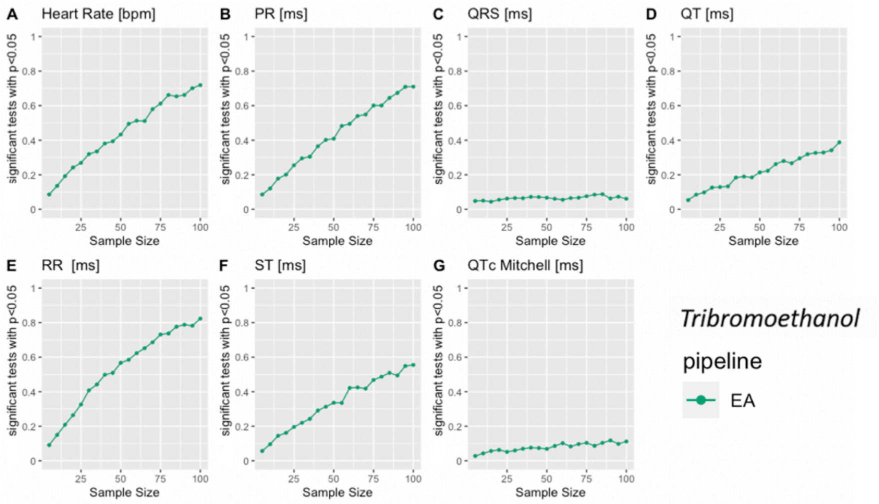

Supplemental Figure 6:

a FEMALE

	p value	Cohen's D
Heart Rate [bpm]	p<.001	-0.49 (small)
PR [ms]	p=.071	0.06 (negligible)
QRS [ms]	p<.001	0.17 (negligible)
QT [ms]	p=.018	0.16 (negligible)
RR [ms]	p<.001	0.46 (small)
ST [ms]	p=.294	0.17 (negligible)
QTc Mitchell [ms]	p=.158	-0.11 (negligible)

b MALE

	p value	Cohen's D
Heart Rate [bpm]	p<.001	-0.38 (small)
PR [ms]	p=.803	0.01 (negligible)
QRS [ms]	p<.001	0.16 (negligible)
QT [ms]	p<.001	0.31 (small)
RR [ms]	p<.001	0.36 (small)
ST [ms]	p<.001	0.42 (small)
QTc Mitchell [ms]	p=.589	-0.04 (negligible)

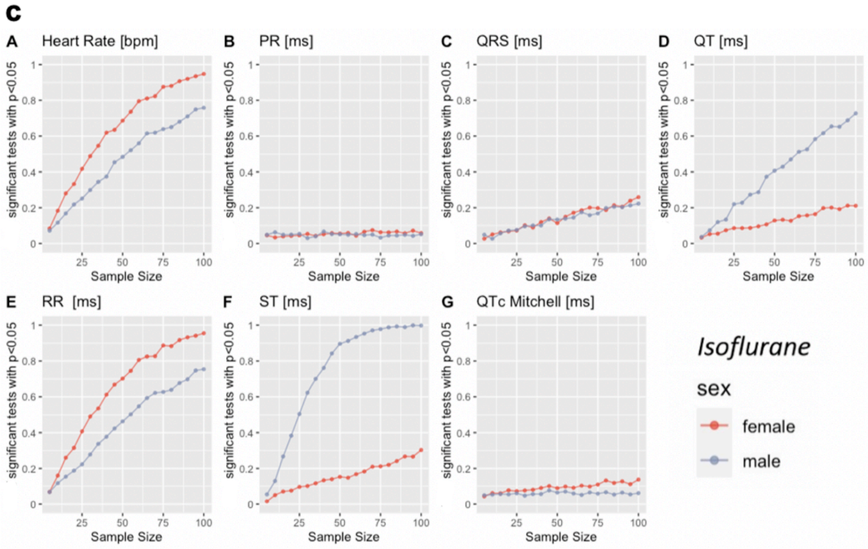

Supplemental Figure 7:

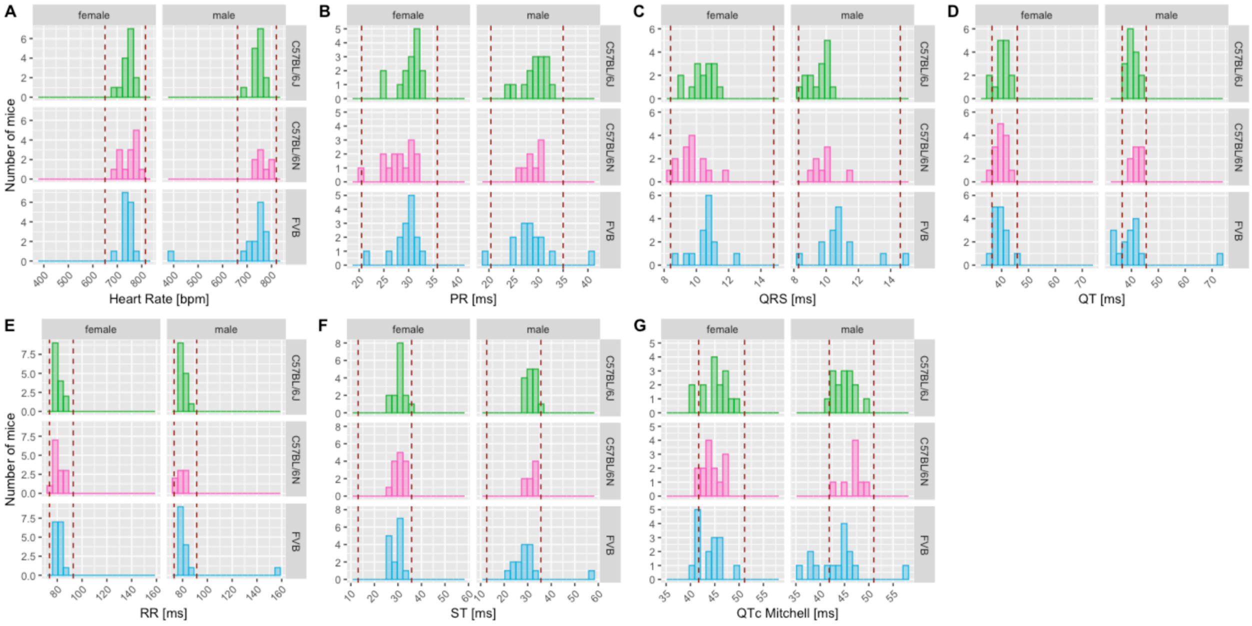

Supplemental Figure 8:

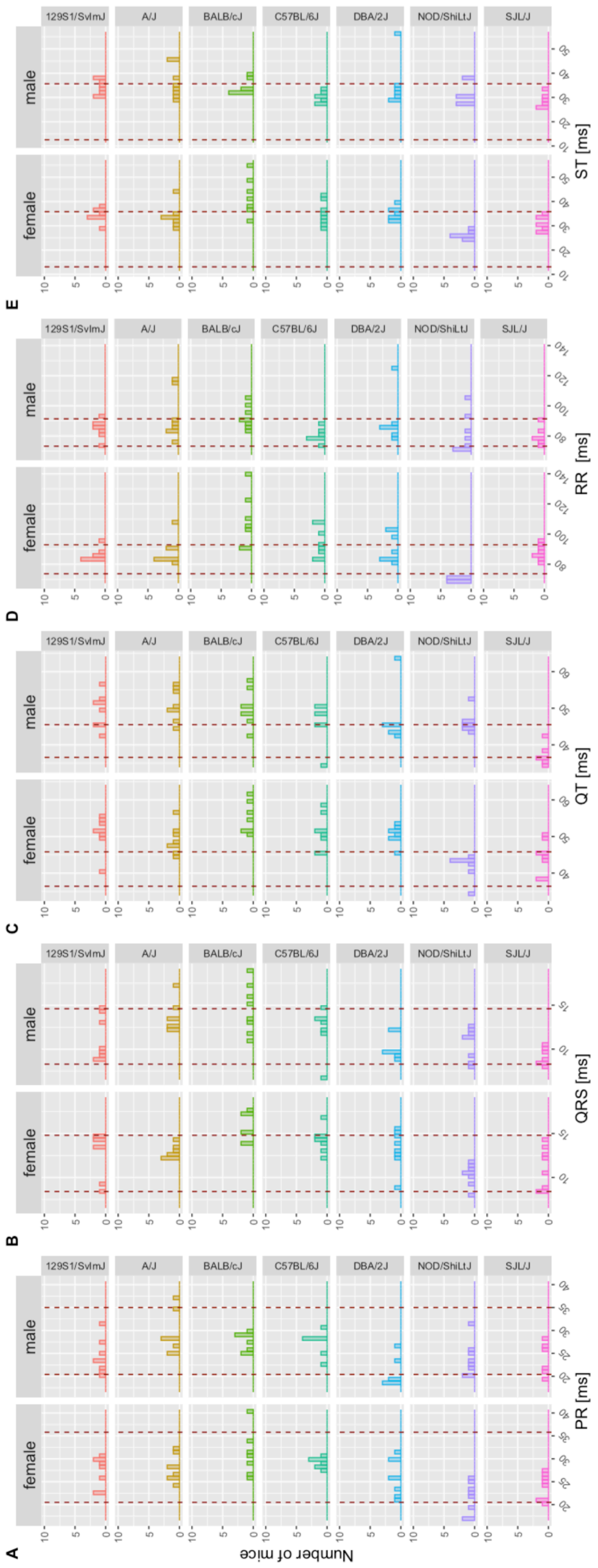

Supplemental Figure 9:

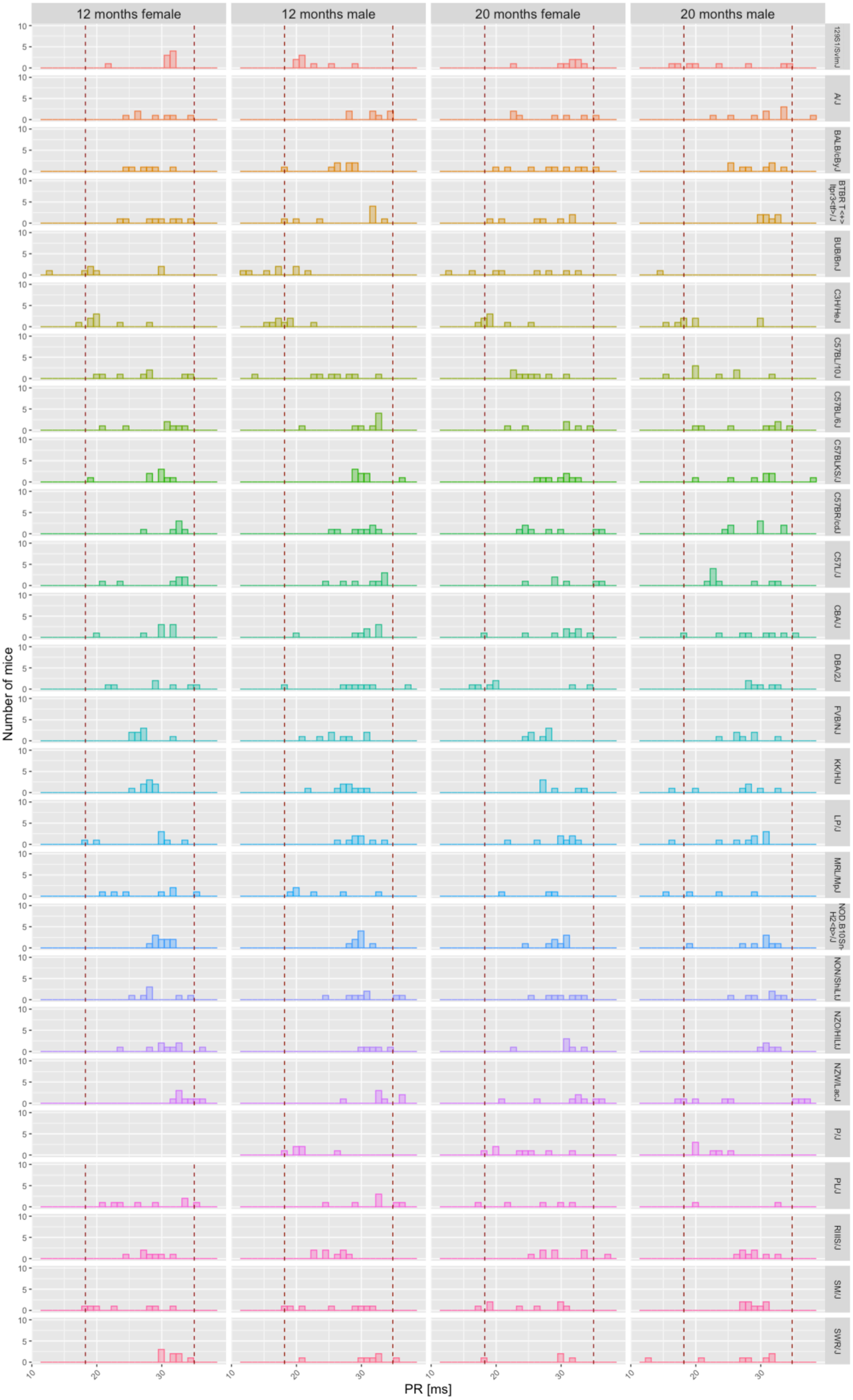

Supplemental Figure 10:

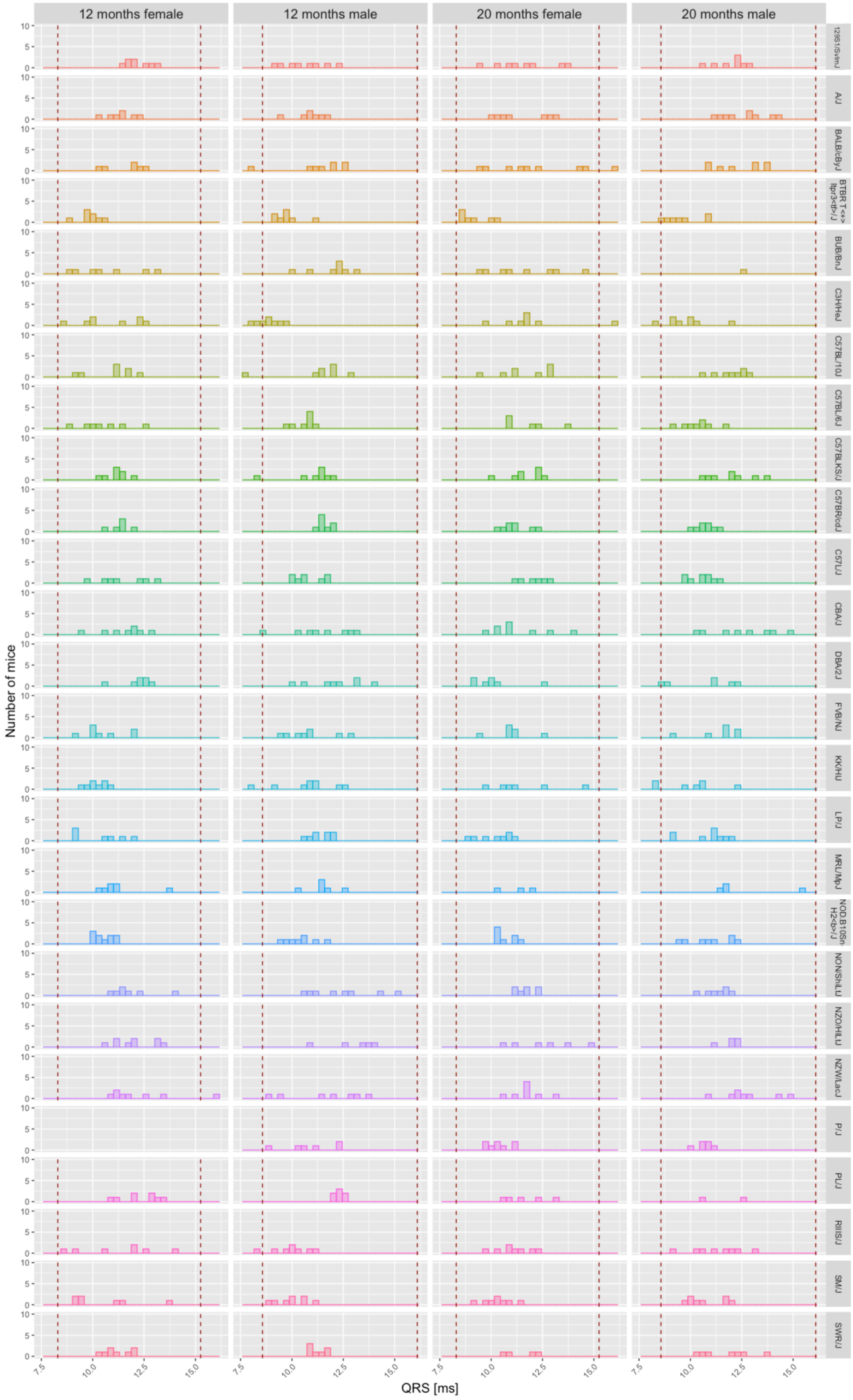

Supplemental Figure 11:

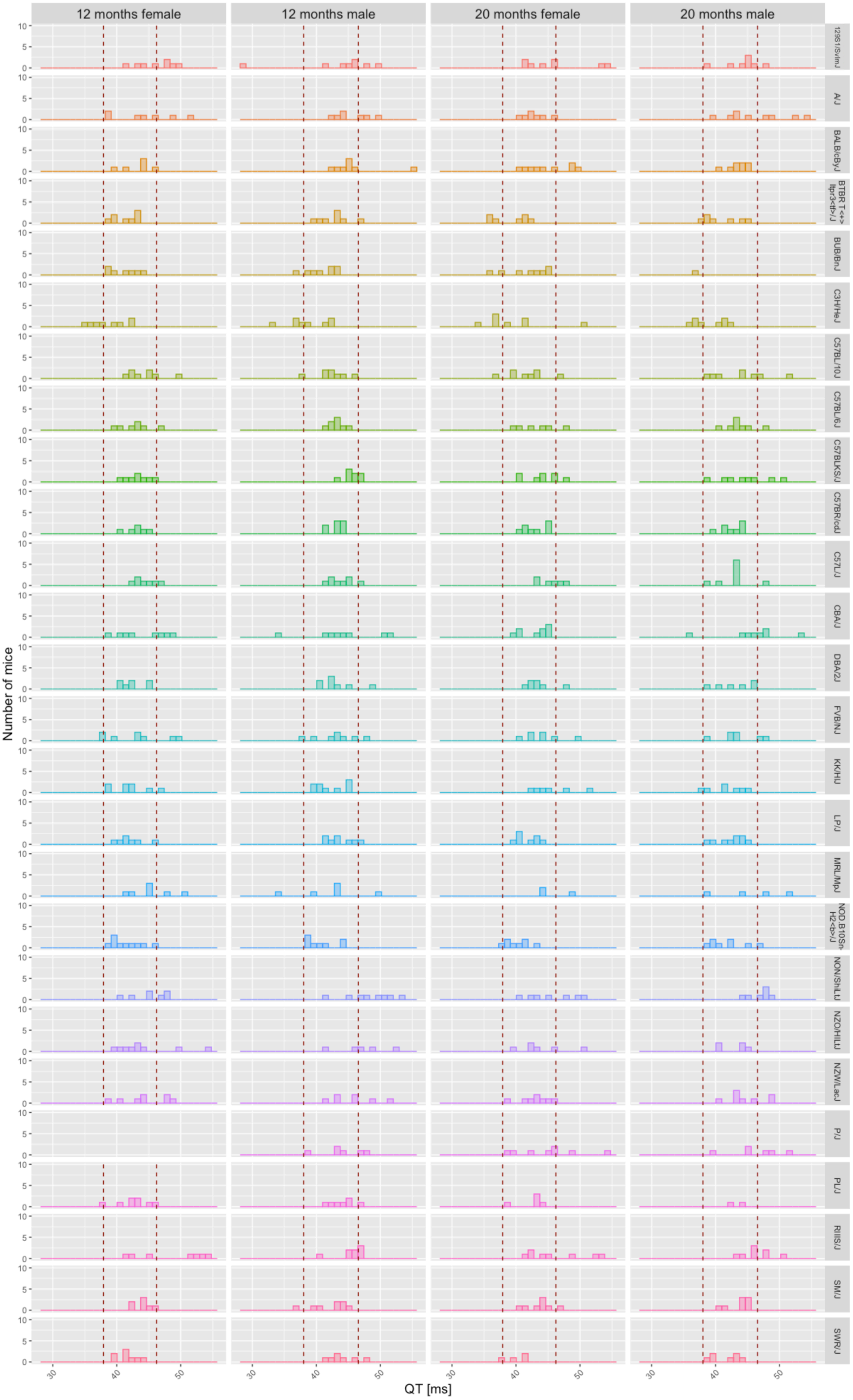

Supplemental Figure 12:

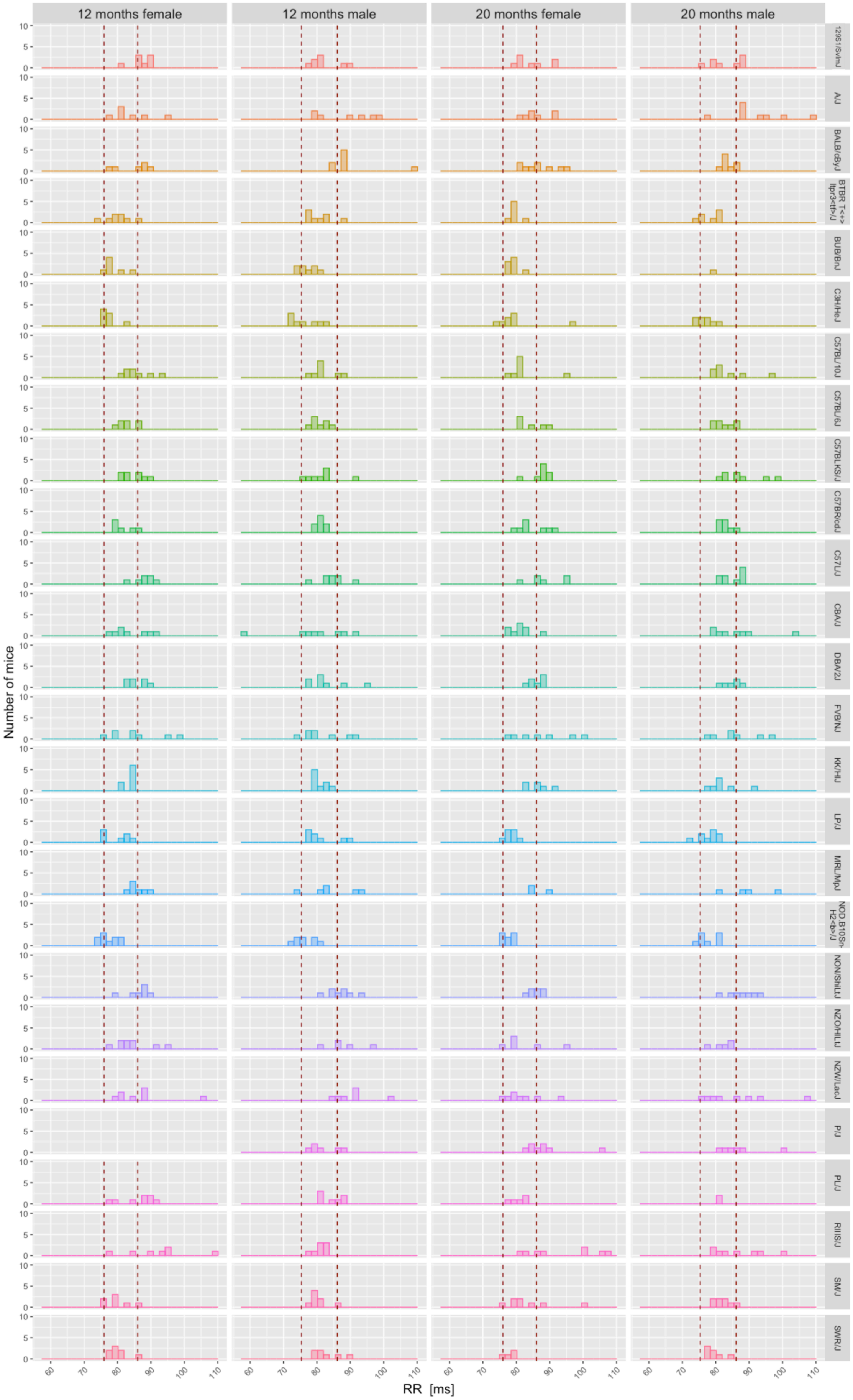

Supplemental Figure 13:

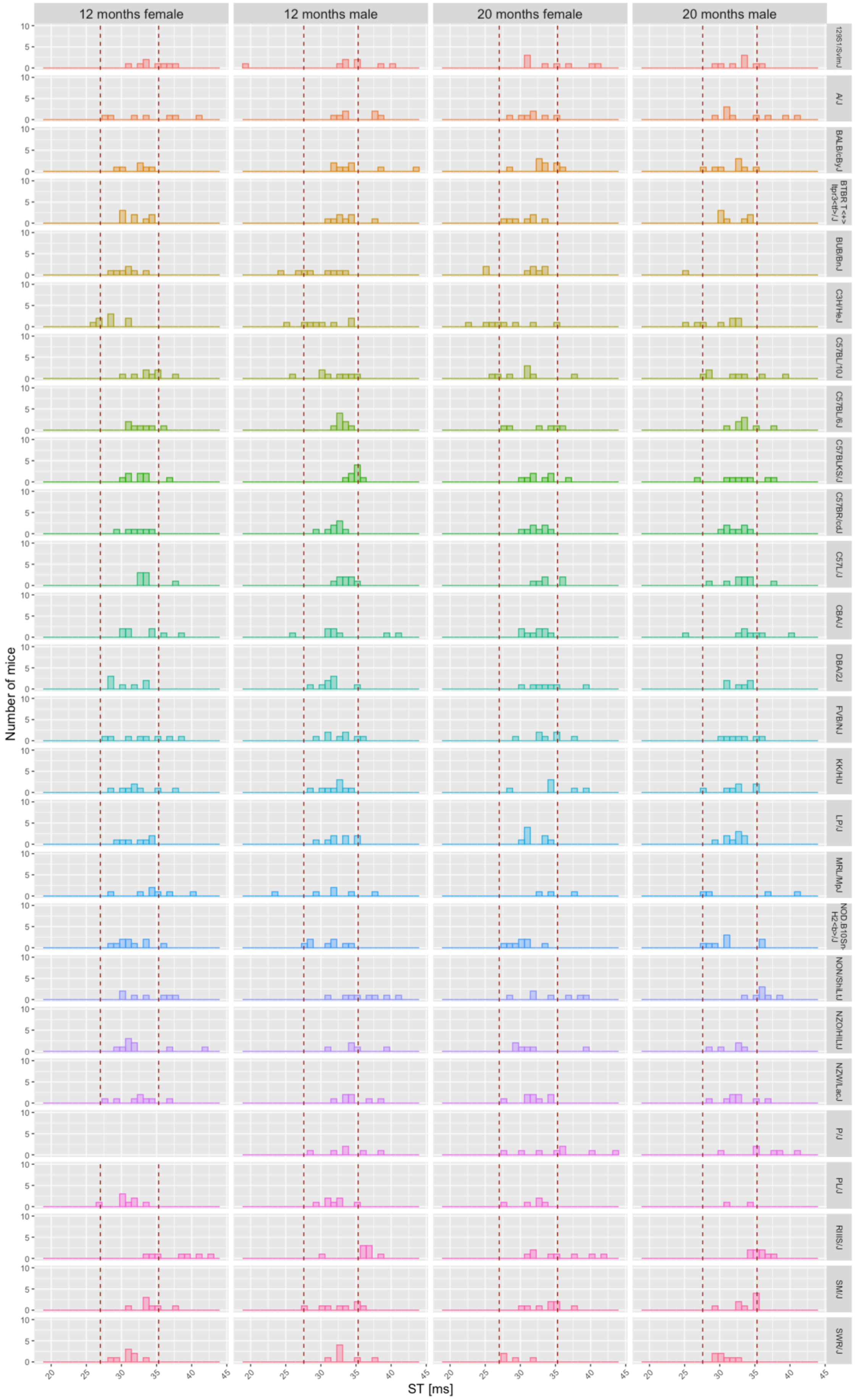

Supplement: Supplementary file 1 — Supplementary file1 (PDF 19886 KB)—Supplemental Table 1: Definition and unit of measure for each ECG parameter reported. Supplemental Table 2: Comprehensive overview of mean, standard deviation, and sample number for each of the seven selected ECG parameters stratified by conscious state (conscious, anesthetized with isoflurane, or anesthetized with tribromoethanol), age (EA and LA timepoint) and sex (Panel a. Females; Panel b. Males). Supplemental Table 3: Comprehensive overview of median and 95% reference ranges (2.5th and 97.5th percentile) as well as mean, standard deviation, and sample number for each of the seven selected ECG parameters, stratified by conscious state (conscious, anesthetized with isoflurane, or anesthetized with tribromoethanol) and age (EA and LA timepoint). Sex is combined (females plus males) for this analysis to generate a both-sex-combined reference range. Supplemental Figure 1: Quartile-based CV (QCV), defined as interquartile range (IQR) (75-25%) relative to the median (100*IQR/median), analysis of data split by sex (female and male) and age (EA and LA) identified parameters with excess variability (QCV ≥30% for EA and LA timepoint) that were excluded from further analysis (white bars). pNN5 could not be calculated due to a zero denominator, therefore it was not displayed. Parameters in blue were below the QCV threshold and were retained for further analysis. In the QCV analysis, but not in the COV, there were three parameters (light blue bars) in the LA population (HR, RR and PR) that marginally exceeded the limit of QCV ≥30 but were retained. Supplemental Figure 2: Histograms presenting the distribution of PQ-interval data along with calculated ranges (mean ± SD and median and 95% reference range) for conscious EA and LA mice stratified by sex. These calculations are based on data from one contributing center (German Mouse Clinic). Supplemental Figure 3: Testing sex-differences in conscious mice. T-test results when comparing data fro [file 335_2023_9995_MOESM1_ESM.pdf]
